# Melanoma-intrinsic NR2F6 activity regulates antitumor immunity

**DOI:** 10.1126/sciadv.adf6621

**Published:** 2023-07-05

**Authors:** Hyungsoo Kim, Yongmei Feng, Rabi Murad, Joanna Pozniak, Carl Pelz, Yeqing Chen, Bhavik Dalal, Rosalie Sears, Eduard Sergienko, Michael Jackson, Eytan Ruppin, Meenhard Herlyn, Curtis Harris, Jean-Christophe Marine, Victoria Klepsch, Gottfried Baier, Ze’ev A. Ronai

**Affiliations:** ^1^ Cancer Center, Sanford Burnham Prebys Medical Discovery Institute, La Jolla, CA, USA.; ^2^ Center for Cancer Biology, VIB, Leuven, Belgium.; ^3^ Department of Molecular and Medical Genetics, Brenden Colson Center for Pancreatic Care, Oregon Health and Science University, Portland, OR, USA.; ^4^ Molecular and Cellular Oncogenesis Program and Melanoma Research Center, The Wistar Institute, Philadelphia, PA, USA.; ^5^ Laboratory of Human Carcinogenesis, Center for Cancer Research, National Cancer Institute, Bethesda, MD, USA.; ^6^ Cancer Data Science Laboratory, National Cancer Institute, National Institutes of Health, Bethesda, MD, USA.; ^7^ Division of Translational Cell Genetics, Medical University of Innsbruck, 6020, Innsbruck, Austria.

## Abstract

Nuclear receptors (NRs) are implicated in the regulation of tumors and immune cells. We identify a tumor-intrinsic function of the orphan NR, NR2F6, regulating antitumor immunity. NR2F6 was selected from 48 candidate NRs based on an expression pattern in melanoma patient specimens (i.e., IFN-γ signature) associated with positive responses to immunotherapy and favorable patient outcomes. Correspondingly, genetic ablation of NR2F6 in a mouse melanoma model conferred a more effective response to PD-1 therapy. NR2F6 loss in B16F10 and YUMM1.7 melanoma cells attenuated tumor development in immune-competent but not -incompetent mice via the increased abundance of effector and progenitor-exhausted CD8^+^ T cells. Inhibition of NACC1 and FKBP10, identified as NR2F6 effectors, phenocopied NR2F6 loss. Inoculation of NR2F6 KO mice with NR2F6 KD melanoma cells further decreased tumor growth compared with NR2F6 WT mice. Tumor-intrinsic NR2F6 function complements its tumor-extrinsic role and justifies the development of effective anticancer therapies.

## Teaser

NR2F6 activity in melanoma impacts its response to anti-tumor immunity.

## INTRODUCTION

The importance of the tumor microenvironment (TME) for cancer development has gained critical traction as we acquire insight into cross-talk between TME components, including immune cells, cancer-associated fibroblasts, endothelial cells (i.e., stroma), and tumors (*1*–*3*). These interactions rely, in part, on signals derived from tumor cells (tumor-intrinsic), while tumor-extrinsic changes in TME components are regulated by cytokines and chemokines secreted by tumors or TME components (*4*–*6*). These factors govern the recruitment of stromal cells and their infiltration of a tumor, which define tumor fate. Our understanding of tumor/TME cross-talk has driven the development of therapeutic modalities and contributed to the evolution of immune checkpoint therapies (ICTs). ICTs target a natural gatekeeping brake, which evolved to prevent self-attack and maintain immune homeostasis and has revolutionized cancer treatment. Blocking an immune checkpoint via ICT often revitalizes the immune system’s ability to eradicate tumor cells (*7*, *8*). ICT is now the first-line therapy for several cancers, as reflected in the growing number of U.S. Food and Drug Administration–approved ICT drugs capable of producing durable tumor remission (*9*). Despite these advances, success is still limited, as a sizable percentage of patients are either nonresponsive or develop resistance to ICT (*3*, *10*, *11*).

Given these limitations, efforts are underway to understand ICT responsiveness mechanisms better and identify biomarkers predictive of a positive ICT response (*12*–*15*). Thus far, responsiveness to ICT is primarily determined by a tumor’s mutation burden, the ability of T cells to infiltrate a tumor, and tumor responsiveness to interferon-γ (IFN-γ) (*16*–*19*). Thus, identifying regulatory components that could be targeted to improve ICT effectiveness and durability remains an important goal and an unmet clinical need.

Factors that define tumor responsiveness to therapies, including ICT, often influence TME activity. Tumor-intrinsic activities can affect the extracellular matrix, as well as stromal and immune components or regulate interactions among TME components (*4*–*6*). In addition to the chemokines and cytokines noted above, nuclear hormone receptors (NRs) also modulate tumor cell communication with the TME and may underlie a tumor’s response to external stimuli (including therapy) or microenvironment [nutrient and oxygen availability; (*20*–*23*)].

In normal cells, NRs reportedly control regulatory pathways that govern proliferation, metabolism, specialized cell functions, and immune cell activities. Likewise, NRs are often deregulated or dysfunctional in pathophysiological conditions, including cancers. Accordingly, NRs are implicated in hormone-dependent cancers (such as breast and prostate cancers) and often serve as markers for patient stratification or as treatment targets (*20*, *21*).

Recent studies also indicate an emerging role for NRs in antitumor immunity. Activation of liver X receptor, for example, induces apolipoprotein E expression within the melanoma niche to attenuate the activity of innate myeloid-derived suppressor cells (MDSCs), resulting in more effective inhibition of metastasis (*24*, *25*). Conversely, tumor-derived retinoic acid reportedly induces monocyte differentiation to immune-suppressive tumor-associated macrophages (TAMs), which dampens ICT impact (*26*). Thus, the possibility of controlling NR activity via regulatory ligands or agonists/antagonists has prompted efforts to identify NRs that modulate the TME and could be exploited in more effective immunotherapy strategies.

The orphan nuclear receptor NR2F6 (nuclear receptor subfamily 2 group f member 6), also known as Ear-2 or Chicken Ovalbumin Upstream Promoter-Transcription Factor III (COUP-TFIII), is a member of the NR2F subfamily and structurally related to NR2F1 and NR2F2 proteins (*27*). In immune cells, NR2F6 has been suggested to be an immune checkpoint candidate based on analysis of its capacity to fine-tune adaptive immunity and repress transcription of genes encoding interleukin-2 (IL-2), IFN-γ, IL-17, and IL-21 cytokines (*27*–*30*). Accordingly, a genetic mouse model with global NR2F6 ablation exhibits accelerated inflammation, autoimmune phenotypes, and attenuated tumor growth due to enhanced activity of tumor-infiltrating effector T cells (*27*, *30*–*33*). These findings were derived from analysis of immune cells; by contrast, the tumor-intrinsic NR2F6 function is less well understood. NR2F6 expression in tumors has been linked to protumorigenic phenotypes, including proliferation, invasiveness, poor prognosis, and resistance to therapy in colon, ovarian, lung, liver, and breast cancers (*34*–*40*). However, the tumor-intrinsic function of NR2F6 in shaping an antitumor immune response in the tumor niche remains largely unexplored.

Here, we show that tumor-intrinsic NR2F6 expression in melanoma cells may mediate immune evasion by controlling the expression of genes that suppress antitumor immunity. Correspondingly, higher NR2F6 expression in melanoma patient specimens was associated with a less favorable prognosis and a poor response to ICT. These findings could affect ICT strategies and provide a basis to stratify patients for therapy based on NR2F6 expression and justification for systemic targeting of NR2F6 as treatment.

## RESULTS

### Identification of NRs that govern antitumor immune responses

**Fig. 1.**
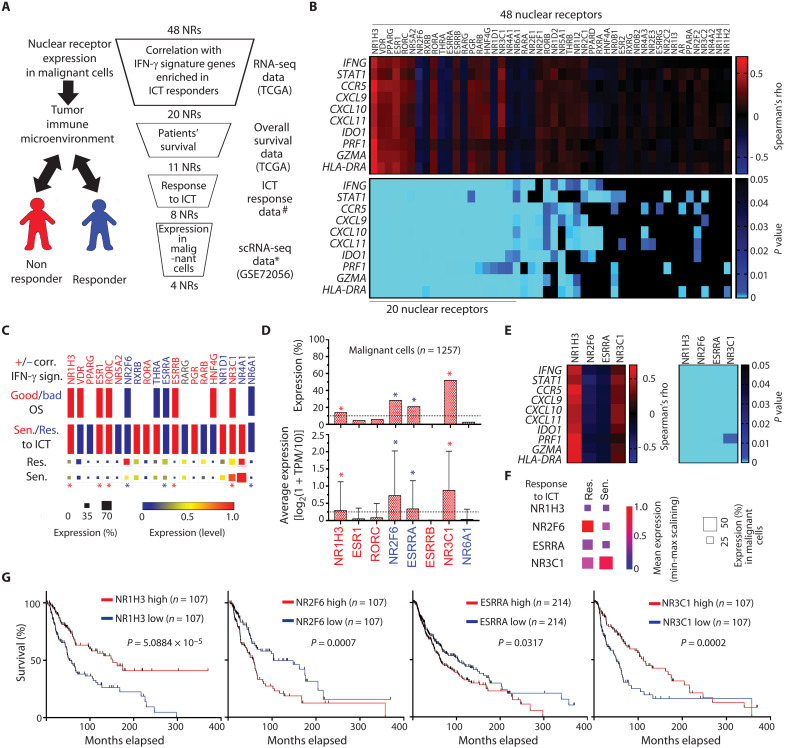
Identification of NRs potentially linked to antitumor immunity in patients with melanoma. (**A**) Outline of workflow in the selection process. ICT response data were obtained from scRNA-seq of patients with melanoma (*59*). * denotes the gene expression data of malignant cells prepared based on GSE70256 (*57*). # denotes *P* < 0.05, Kaplan Meier analysis. (**B**) Heatmaps show Spearman’s correlation coefficient (top) and corresponding *P* values (bottom) in comparisons of expression of 48 NRs (*x* axis) with 10 IFN-γ signature genes (*y* axis). Twenty NRs showing a significant correlation with the IFN-γ signature were selected. (**C**) Those 20 NRs, which were either positively (red) or negatively (blue) correlated (Corr.) with the IFN-γ signature (IFN-γ sign.), were assessed for correlation with patient overall survival (top) and patient responses to ICT (bottom). Relevant to overall survival, red and blue bars represent respective favorable and unfavorable overall survival relative to the expression of corresponding NRs. Red and blue bars are relevant to ICT response, representing the respective “sensitivity” and “resistance” of patients with high NR-expressing tumor cells to ICT. Squares at the bottom show percent expression and expression level of NRs in tumor cells from patients resistant (Res, top) or sensitive (Sen, bottom) to ICT. Expression of eight NRs that correlated with patient response to ICT, were selected (indicated by asterisks). (**D**) Tumor-intrinsic average and percent expression of those eight NRs was assessed using scRNA-seq data from specimens of patients with melanoma (*57*). Four NRs (as indicated by asterisks) expressed at higher levels in >10% of tumor cells were either positively or negatively correlated with IFN-γ signature genes (**E**), response to ICT (**F**), or overall patient survival (**G**). TCGA, The Cancer Genome Atlas; OS, overall survival; TPM, transcript per million.

To identify factors that control ICT responses, we searched for NRs that may regulate antitumor immunity. To this end, transcriptome data from bulk and single-cell RNA sequencing (scRNA-seq) analysis, as well as clinical information on patients’ survival and response to ICT, were assessed. Initially, we focused on the transcriptomic data to identify NRs whose expression correlated either positively or negatively with an immune-responsive gene signature. Selected NRs were further assessed on the basis of clinical outcomes (Fig. 1A).

Among the top-ranked genes identified in the NanoString analysis of genes associated with melanoma responsiveness to ICT and its microenvironment (including immune-infiltrated cells) was a cluster of 10 genes implicated in the IFN-γ response [*IFNG*, *STAT1*, *CCR5*, *CXCL9*, *CXCL10*, *CXCL11*, *IDO1*, *PRF1*, *GZMA*, and *HLA-D*; (*16*)]. IFN-γ signaling is a well-established immune-responsive gene signature enriched in samples from patients with melanoma that exhibit a positive response to ICT and better overall survival (*16*). We thus asked whether the expression of any of the 48 candidate NRs correlated with this signature and identified 20 that showed either a positive or negative correlation (Fig. 1B). On the basis of the strong positive correlation between the IFN-γ-signature and patients’ clinical outcomes [fig. S1A; (*16*)], we asked which of the 20 candidates correlated with patient survival and ICT responses. This led us to the identification of 11 genes showing significant positive or negative correlations with patient outcomes (Fig. 1C, upper bars for “Good/Bad OS”). Of those, eight were significantly correlated (positively or negatively) with an ICT response: Patients whose specimens highly expressed one of five NRs (NR1H3, NR3C1, RORC, ESR1, or ESRRB) exhibited a better response to ICT, while high expression of either NR2F6, NR6A1, or estrogen-related receptor alpha (ESRRA) was associated with resistance to ICT (Fig. 1C, lower bars). Given that NRs are expressed in numerous cell types in the tumor niche, we focused on NRs expressed in tumor cells that may modulate immune evasion or antitumor immunity. To this end, we assessed NR expression in malignant melanoma cells within the tumor niche using single-cell transcriptomic data. Among NRs expressed in >10% of melanoma cells were NR1H3, NR3C1, NR2F6, and ESRRA (Fig. 1D). All four were highly expressed in malignant cells (13 to 51%) and either positively (NR2F6 and ESRRA) or negatively (NR1H3 and NR3C1) correlated with the IFN-γ-signature (Fig. 1E), responses to ICT (Fig. 1F), and overall patient survival (Fig. 1G). Three of these genes (ESRRA, NR1H3, and NR3C1) were also expressed in multiple immune cell types in the TME (fig. S1B), while NR2F6 was expressed primarily in melanoma and nonimmune stromal cells, including endothelial cells and cancer-associated fibroblasts (fig. S1C).

### NR2F6 control of murine melanoma growth requires an intact immune system

**Fig. 2.**
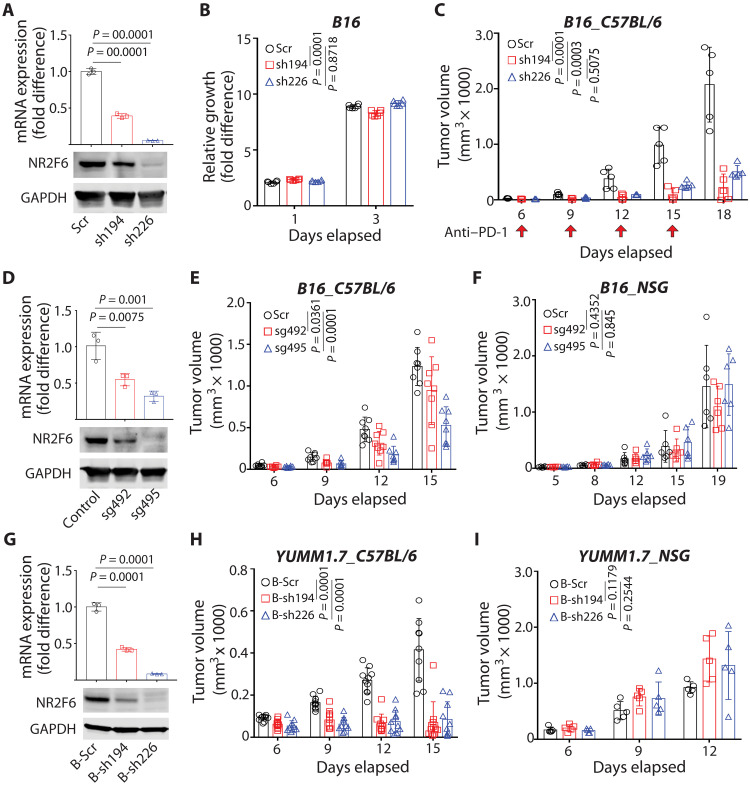
NR2F6 control of murine melanoma growth requires an intact immune system. (**A**) B16F10 cells were transduced with scramble (Scr) control shRNA or one of two shRNAs (sh194 and sh226) targeting murine *Nr2f6*. NR2F6 mRNA and protein expression was assessed by qPCR and immunoblotting. *n* = 3 for each group. GAPDH, glyceraldehyde-3-phosphate dehydrogenase. (**B**) Growth of cultured cells described in (A), as assessed in vitro using CellTiter-Glo. Relative fold differences in luminescence on days 1 and 3 were calculated relative to luminescence on day 0. *n* = 6 for each group. (**C**) C57BL/6 mice were then inoculated with cells and treated with anti–PD-1 antibody (RMP1-14) on days 6, 9, 12, and 15 (arrows). Tumor volumes were monitored at indicated time points. *n* = 5 mice for each group. (**D**) NR2F6 KO B16F10 cells were established by CRISPR using specific single guide RNAs (sg492 and sg495). NR2F6 mRNA and protein expression was assessed by qPCR and immunoblotting, respectively. *n* = 3 for each group. (**E** and **F**) Cells established in (D) were used to inoculate C57BL6 (E) or NSG (F) mice, and tumor volumes were monitored at indicated time points. *n* = 8 mice (E) and *n* = 6 mice (F) for each group. (**G**) YUMM1.7 cells were transduced with scrambled (B-Scr) control shRNA or one of two shRNAs (B-sh194 and B-sh226) targeting *Nr2f6* as in (A). mRNA and protein expression was assessed by qPCR and immunoblotting, respectively. *n* = 3 for each group. (**H** and **I**) Cells established in (G) were then used to inoculate C57BL/6 (H) or NSG (I) mice, and tumor volumes were monitored at indicated time points. *n* = 9 mice (H) and *n* = 5 mice (I) for each group. Data are presented as means ± SD. Statistical significance was assessed by one-way analysis of variance (ANOVA) with Dunnett’s test (A, D, and G) and two-way ANOVA with Dunnett’s test (B, C, E, F, H, and I).

We next asked whether altering the expression of any of the four candidate NRs in melanoma cells would change melanoma responses to ICT using anti–programmed cell death protein 1 (anti–PD-1) antibodies (RMP-14 clone) as a checkpoint blockade. To this end, we first confirmed that the four candidate NRs were expressed in mouse melanoma lines. Mouse melanoma line B16F10, which is also unresponsive to ICT (*14*), expressed higher levels of NR2F6 and ESRRA and lower levels of NR1H3 and NR3C1 than did other mouse melanoma lines (fig. S2A). Given that NR2F6 and/or ESRRA expression was inversely correlated with the IFN-γ signature (Fig. 1E), we used short hairpin RNA (shRNA) to engineer two types of B16F10 cell lines, either NR2F6 or ESRRA knockdown (KD) lines or NR1H3 or NR3C1 overexpression (OE) lines (Fig. 2A and fig. S2B) and assessed potential growth changes in cultures of each. Cultured cells overexpressing NR1H3 or NR3C1 or deficient in NR2F6 showed comparable growth rates (Fig. 2B and fig. S2C). By contrast, ESRRA KD slowed B16F10 cell growth in culture, consistent with observations made in ESRRA-depleted human melanoma cells [DepMap (depmap.org/portal/); fig. S2D]. We then inoculated C57BL/6 mice with cells from NR1H3 or NR3C1 OE, NR2F6 KD, or control wild-type (WT) B16F10 melanoma lines; treated animals with anti–PD-1 antibodies; and evaluated them for responses to ICT. NR1H3 or NR3C1 OE in tumor cells in vivo had no notable effect on ICT responsiveness (fig. S2E), while NR2F6 KD notably delayed melanoma growth in mice treated with anti–PD-1 antibodies (Fig. 2C), suggesting that NR2F6 loss converts B16F10 cells from an immunologically cold to warm status to allow a response to ICT.

To further assess how NR2F6 expression affects antitumor immunity, we compared the growth of NR2F6-deficient B16F10 cells in immune-incompetent versus immune-competent mice. Notably, compared with NR2F6 WT B16F10 cells, NR2F6 KD via either one of two shRNAs (Fig. 2A) led to 43.2% (sh226) or 68.7% (sh194) inhibition of B16F10 melanoma growth in immune-competent (C57BL/6) but not immune-incompetent [Nod-Scid-Gamma (NSG)] mice compared to cells harboring WT NR2F6 (fig. S2, F and G). Noting that the degree of tumor growth inhibition did not coincide with the efficiency of NR2F6 KD, we set to confirm these observations by an independent genetic approach. To this end, we genetically ablated NR2F6 using CRISPR in B16F10 cells (Fig. 2D and fig. S2, H and I). Consistent with NR2F6 KD results, CRISPR-mediated NR2F6 knockout (KO) inhibited tumorgrowth by 23.5% (sg492) to 44.1% (sg495) in immune-competent (C57BL/6) but not immune-incompetent (NSG) mice (Fig. 2, E and F). Likewise, NR2F6 ablation in the YUMM1.7(*Braf^V600E^/Pten^−/−^/CDKN2A^−/−^*) melanoma line attenuated tumor formation in immune-competent but not immune-incompetent mice (Fig. 2, G to I, and fig. S2J). These findings suggest that antitumor phenotypes seen following NR2F6 loss in tumors require an intact host immune system. Given that tumor-intrinsic NR2F6 loss attenuates B16F10 cell growth, we asked whether ectopic NR2F6 expression would enhance tumor development. To do so, we inoculated mice with B16F10 cells overexpressing either WT or a DNA binding-defective (C112S) NR2F6 mutant [fig. S2, K and L; (*41*)] and monitored tumor growth. While WT NR2F6 OE did not alter tumor growth, expression of the DNA binding-defective mutant, which should mimic NR2F6 KD, attenuated tumor growth in mice and extended overall animal survival (fig. S2, M and N), suggesting that NR2F6 transcriptional activity is required, but ectopic OE of NR2F6 only is not sufficient to accelerate tumor growth.

### Tumor cell–intrinsic NR2F6 expression controls T cell infiltration

**Fig. 3.**
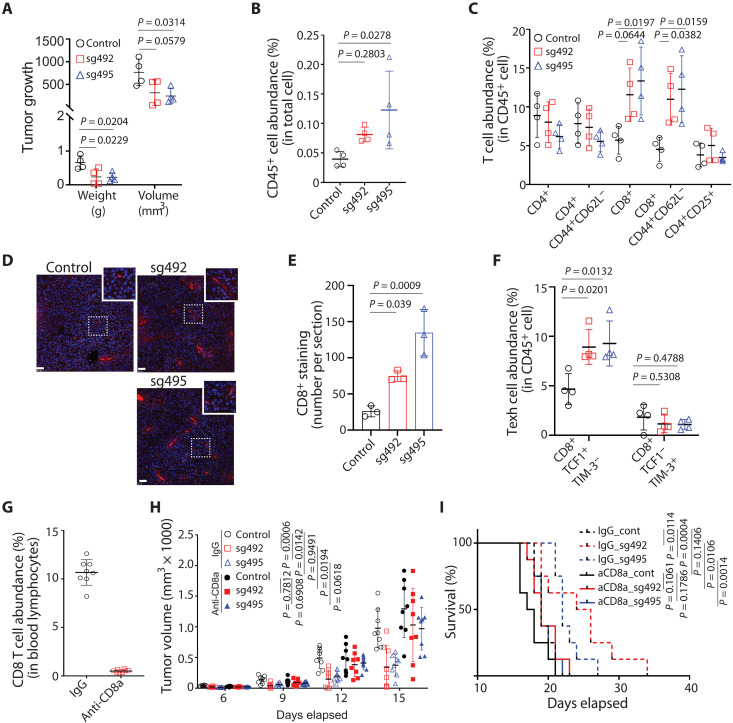
NR2F6 limits murine melanoma growth by controlling CD8 T cell infiltration. (**A**) Control and NR2F6 KO B16F10 cells were engrafted into C57BL/6 mice. Tumors were collected 12 days later and assessed for weight and volume. *n* = 4 mice for each group. (**B**) CD45^+^ cell abundance (expressed as a percentage) in cells within the singlet gate, as assessed by FACS. *n* = 4 mice for each group. (**C**) Abundance of T cell subtypes within all CD45^+^ cells, as assessed by fluorescence-activated cell sorter (FACS). *n* = 4 mice for each group. (**D** and **E**) Control and NR2F6 KO (sg492 and sg495) B16F10 tumor sections were prepared from tumors collected on day 18 after inoculation and stained with anti-mouse CD8 antibody. The number of CD8^+^ staining was visualized via microscope (×20) and counted in four random regions of each section. *n* = 3 mice for each group. Scale bar, 50μm. (**F**) Control and NR2F6 KO B16F10 tumors were prepared as in (A). FACS assessed the abundance of Texh subsets. *n* = 4 mice for each group. (**G** to **I**) Mouse groups were injected with control IgG or anti-CD8A antibodies to deplete CD8^+^ T cells. Eight days later, CD8^+^ T cell abundance was assessed in blood samples (G). Tumor growth (H) and overall animal survival (I) were monitored at indicated time points. *n* = 8 mice for each group. Data are presented as means ± SD. Statistical significance was assessed by one-way ANOVA with Dunnett’s test (A, B, C, E, and F), Student’s *t* test (G), two-way ANOVA with Sidak’s test (H), or by long-rank test (I).

To further assess the effects of tumor-intrinsic NR2F6 expression on antitumor immunity, we monitored the infiltration of melanoma tumors by tumor-infiltrating lymphocytes (TILs) in the presence or absence of tumor-intrinsic NR2F6 expression. To do so, we engineered NR2F6-deficient B16F10 tumor cells, injected them into immune-competent mice, and collected tumors 12 days later. As expected, NR2F6 loss reduced tumor volume and weight compared with NR2F6 WT controls (Fig. 3A). Notably, NR2F6-ablated tumors exhibited a 1.5- to 2-fold increase in the number of infiltrating CD45^+^ immune cells (Fig. 3B), an increase more pronounced in the population of effector memory CD8^+^ T cells (2- to 2.5-fold increase in CD8^+^CD44^+^ CD62L^−^ cells; Fig. 3C and fig. S3A). Consistent with this observation, NR2F6 depletion by CRISPR KO or shRNAs increased CD8^+^ T cell staining in B16 (Fig. 3, D and E) and YUMM1.7 (fig. S3B) tumors. NR2F6-deficient tumors exhibited increased IFN-γ production and PD-1 expression, which was not statistically significant (fig. S3, C and D). Given the importance of a subset of exhausted CD8 cytotoxic T lymphocytes (CTLs) designated “progenitor exhausted” in control of tumor growth and the ICT response (*42*), we assessed the abundance of progenitor exhausted (CD8^+^Tcf1^+^Tim3^−^) versus “terminally exhausted” (CD8^+^Tcf1^−^Tim-3^+^) CTLs in NR2F6 WT and KO tumors (fig. S3F). Notably, NR2F6 KO tumors exhibited significantly greater numbers of progenitor exhausted CD8^+^ T cells than WT tumors, an effect not seen in terminally exhausted T cells (Fig. 3F), suggesting that NR2F6 deficiency increases the abundance of functionally intact subset of exhausted CD8^+^ T cells that can better control tumor growth. By contrast, the abundance of other immune cells, except for B220^+^ B cells, was unchanged by tumor cell NR2F6 depletion (fig. S3E). In further analyses of myeloid immune cells, NR2F6 KO tumors did not show significantly increased infiltration by M1 (CD11b^+^F4/80^+^CD38^+^EGR2^−^) or M2 (CD11b^+^F4/80^+^CD38^−^EGR2^+^ or CD11b^+^F4/80^+^CD38^−^CD206^+^) TAMs (*43*) or by cDC1 (CD11c^+^MHCII^+^CD8a^+^XCR1^+^) or M-MDSC (CD11b^+^Gr1^+^Ly6C^+^Ly6G^−^) cells (fig. S3, F to I). These observations suggest that increased infiltration of functionally intact CD8^+^ CTLs appears to be the most pronounced change in antitumor immunity following NR2F6 deficiency.

To confirm that CD8^+^ T cell infiltration mediates tumor regression after NR2F6 loss, we depleted CD8^+^ T cells from mice inoculated with NR2F6-deficient B16F10 cells via injection of anti-CD8 neutralizing antibodies (Fig. 3G). Unlike NR2F6 KO tumor-bearing mice treated with immunoglobulin G (IgG) antibodies, CD8^+^ T cell depletion abolished tumor growth inhibition and decreased survival of mice harboring NR2F6 KO B16F10 tumors (Fig. 3, H and I). Notably, CD8^+^ T cell depletion did not fully rescue the growth of NR2F6 KO tumors to the degree seen in control NR2F6 WT tumors, suggesting that increased antitumor immunity seen in NR2F6-deficient melanoma is predominantly, but not solely, mediated by tumor-infiltrating CD8^+^ T cells.

### RNA-seq identifies NACC1 and FKBP10 as NR2F6 effectors

**Fig. 4.**
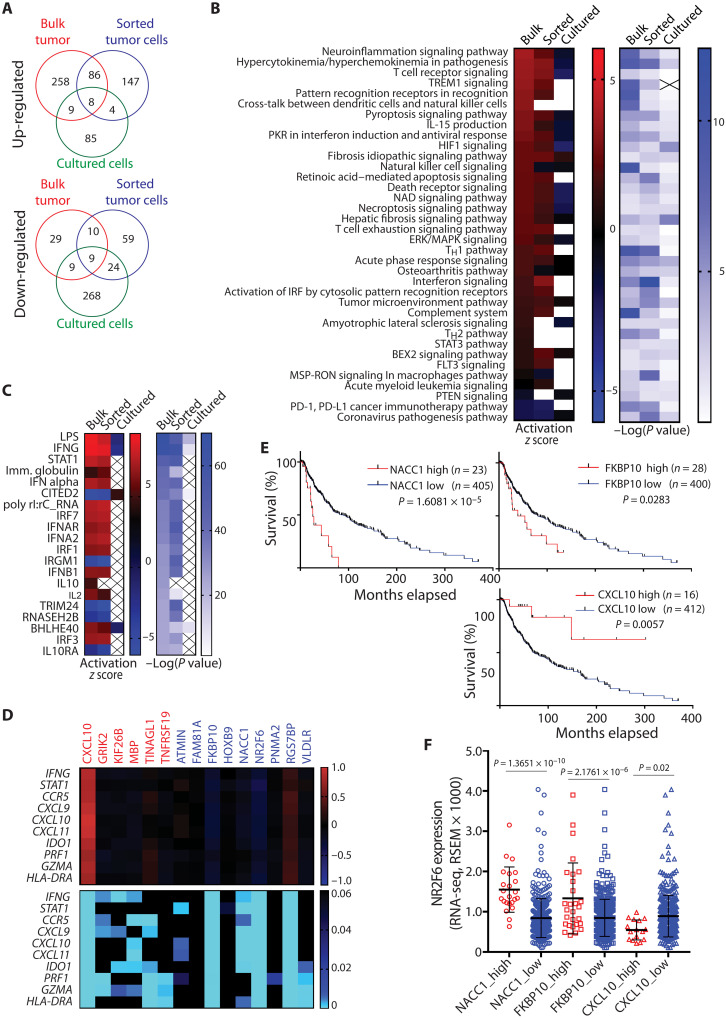
RNA-seq identifies NACC1 and FKBP10 as NR2F6 effectors. (**A**) DEGs between control and CRISPR-KO B16F10 were identified on the basis of RNA-seq analysis of bulk tumors, MACS-sorted tumor cells, or cultured cells. Unique or common genes up-regulated (top) or down-regulated (bottom) are plotted in Venn diagrams. (**B** and **C**) Activated or repressed pathways (B) and upstream regulators (C) of identified DEGs were analyzed using Ingenuity Pathway Analysis. (**D**) Heatmaps show the Spearman’s correlation coefficient (top) and corresponding *P* values (bottom) in expression analysis of 15 genes commonly up- or down-regulated in RNA-seq data from the three samples described in (A). *x* and *y* axes indicate up-/down-regulated genes and 10 IFN-γ signature genes, respectively. CXCL10, FKBP10, and NACC1 expression were significantly correlated with NR2F6 and the IFN-γ signature. (**E**) Overall survival of melanoma patients with relatively high expression (*z* score > 2.0) of NACC1, FKBP10, or CXCL10 was compared with all other patients (*z* score < 2.0) based on Kaplan-Meier analysis. (**F**) NR2F6 expression was assessed in melanoma specimens from patients with low or high expression of NACC1, FKBP10, or CXCL10 in (E). Data are presented as means ± SD. Statistical significance was assessed by log-rank test (E) or one-way ANOVA with Sidak’s test (F). ERK, extracellular signal–regulated kinase; MAPK, mitogen-activated protein kinase; HIF1, hypoxia-inducible factor 1; IRF, interferon regulatory factor; T_H_1, T helper 1; FLT3, fms-like tyrosine kinase 3; BEX2, brain-expressed X-linked 2; PD-L1, programmed death-ligand 1; LPS, lipopolysaccharide; NAD, nicotinamide adenine dinucleotide.

Next, we used RNA-seq to assess transcriptomic changes in the presence of NR2F6 KO and WT tumor cells. To distinguish between changes in tumors versus infiltrating TME components, we performed RNA-seq analysis of bulk tumor samples, magnetic cell sorter (MACS)–sorted tumor cells (to eliminate stromal cells), and cultured B16F10 melanoma cells. Principal components analysis of RNA-seq data indicated distinct gene expression patterns in NR2F6 KO versus control samples (fig. S4A). Notably, the use of sg495 [one of the two single guide RNAs (sgRNAs) used for NR2F6 KO] resulted in better separation of tumor from control samples (fig. S4B), and thus we focused on this sample set. The analysis of bulk B16F10 tumors (in vivo grown), sorted B16F10 tumor cells (in vivo grown), and cultured B16F10 cells revealed that NR2F6 KO differentially up-regulated 361, 245, and 106 genes and down-regulated 57, 102, and 310 genes, respectively (Fig. 4A and fig. S4C). Consistent with tumor growth inhibition seen upon NR2F6 loss, both pathway and upstream regulator analyses of differentially expressed genes (DEGs) identified activation of antitumor immune signaling only in vivo (in bulk tumor and sorted tumor cell samples). Among pathways up-regulated by NR2F6 KO were inflammation, T cell receptor, and T helper 1, and IFN signaling, while common upstream regulators identified were lipopolysaccharide, IFN-γ stimuli, and stimuli-activating signal transducers and activators of transcription 1 (STAT1) signaling (Fig. 4, B and C). By contrast, we observed no activation of immune-related pathways in cultured melanoma cell samples (Fig. 4, B and C), indicating the influence of in vivo components, including the TME, on immune-related pathways in sorted tumor cells.

Given the inverse correlation of NR2F6 expression with an IFN-γ signature (Fig. 1E) and strong activation of IFN-γ downstream genes (Fig. 4C), we assessed possible direct changes in IFN-γ signaling pathway upon NR2F6 loss. STAT1 phosphorylation, a key indicator of the cellular response to IFN-γ signaling, in B16F10 or YUMM1.7 cells depleted of NR2F6 (by shRNAs or CRISPR) and treated with IFN-γ was unaltered compared to NR2F6 WT (fig. S4, D and E). These findings point to transcriptional, rather than posttranslational, modifications as drivers of phenotypes associated with tumor immune evasion, seen upon NR2F6 inhibition. Likewise, NR2F6 expression was unchanged in cultured human melanoma cells before and after IFN-γ or tumor necrosis factor–α (TNFα) treatment [fig. S4H; (*44*)]. These observations suggest that NR2F6 expression is not subject to a feedforward control mechanism modulated by either IFN-γ or TNFα.

To identify NR2F6 effectors, we next assessed 17 genes up- or down-regulated in NR2F6 KO samples from bulk tumors, sorted, and cultured cells (Fig. 4A) for association with both IFN-γ signature genes and patient survival. Of nine genes down-regulated by NR2F6 loss, NACC1 and FKBP10 were negatively correlated with the IFN-γ signature (Fig. 4D), while high NACC1 and FKBP10 expression was associated with poor overall survival (Fig. 4E), patterns seen upon tumor-intrinsic NR2F6 inactivation. Likewise, CXCL10 up-regulation seen following NR2F6 KO was positively correlated with an IFN-γ signature (Fig. 4D), and high CXCL10 expression was associated with good overall survival (Fig. 4E). Accordingly, NR2F6 expression was positively correlated with NACC1 and FKBP10 expression and negatively with CXCL10 expression in human melanoma cells and tissue specimens (Fig. 4F and fig. S4I).

### NACC1 and FKBP10 loss phenocopies enhanced antitumor immunity seen upon NR2F6 loss

**Fig. 5.**
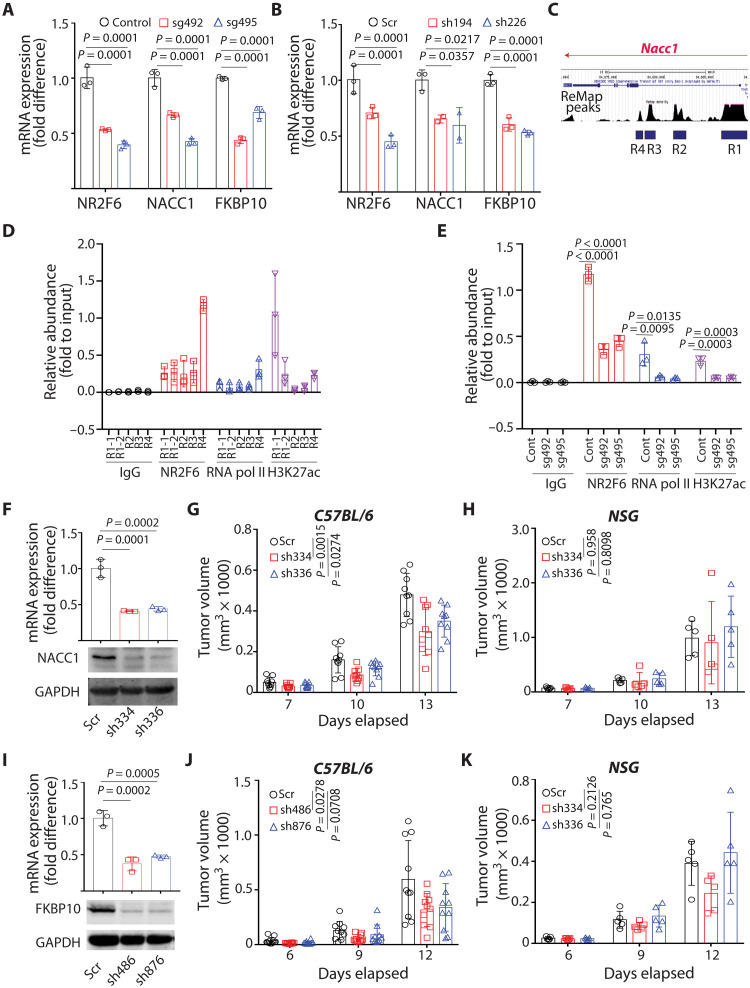
Loss of NACC1 or FKBP10 attenuates tumor growth in mice with an intact immune system. (**A** and **B**) Expression of indicated genes was assessed in NR2F6 KO (CRISPR-based) (A) or KD (shRNA-based) (B) B16F10 cells by qPCR. *n* = 3 for each group. (**C**) ReMap peaks predicted transcriptional regulatory regions of *Nacc1*. R1 included promoter and a part of intron1, and the other candidates (R2, R3, and R4) were predicted within intron1. (**D**) Abundance of NR2F6, RNA polymerase II, and H3K27 acetylation on each candidate region was assessed by ChIP-qPCR using corresponding antibodies and primers. Relative abundance to input (5% of pre-pulldown material) was calculated. *n* = 3 for each group. (**E**) Abundance of NR2F6, RNA polymerase II, and H3K27 acetylation on R4 was assessed in control and NR2F6 KO B16F10 cells. *n* = 3 for each group. (**F**) B16F10 cells were transduced with control scrambled (Scr) shRNA or two shRNAs (sh334 and sh336) targeting NACC1. mRNA and protein expression was assessed by qPCR and immunoblotting, respectively. *n* = 3 for each group. (**G** and **H**) Cells were then used to inoculate C57BL/6 (G) or NSG (H) mice. Tumor volumes were monitored at indicated time points. *n* = 9 mice (G) and *n* = 5 mice (H) for each group. (**I** to **K**) As in (F) and (H), B16F10 cells were transduced with control scrambled shRNA or two (sh486 and sh876) shRNAs targeting FKBP10. FKBP10 expression was assessed (I). *n* = 3 for each group. The growth of tumors emerging from transduced cells was monitored in C57BL/6 (J) or NSG (K) mice. *n* = 10 mice (J) and *n* = 5 mice (K) for each group. Data are presented as means ± SD. Statistical significance was assessed by one-way ANOVA with Dunnett’s test (A, B, E, F, and I) or two-way ANOVA with Dunnett’s test (G, H, J, and K).

To further assess candidate NR2F6 effectors, we first validated NACC1, FKBP10, and CXCL10 expression levels in NR2F6 KD or KO mouse melanoma cells. Consistent with RNA-seq analysis, both NACC1 and FKBP10 expression was down-regulated in NR2F6 KD or KO mouse melanoma, B16F10, and YUMM1.7 cells (Fig. 5, A and B, and fig. S5A). However, up-regulated CXCL10 seen in NR2F6-deficient cells based on RNA-seq analysis was not validated in cultured melanoma cells (fig. S5B), and thus we focused on NACC1 and FKBP10.

To determine how NR2F6 regulates NACC1 and FKBP10 expression, we monitored potential direct NR2F6 binding to their regulatory regions using chromatin immunoprecipitation (ChIP) sequencing data-based analyses of mouse *Nacc1* and *Fkbp10* genes (ReMap 2002; see Materials and Methods). This analysis indicated four potential regions (R1 to R4) of NR2F6 binding to *Nacc1* and two (R1 and R2) for *Fkbp10* (Fig. 5C and fig. S5C). Among them, *Nacc1* R4 exhibited strong NR2F6 binding (Fig. 5D), while NR2F6 did not interact with either candidate region of *Fkbp10* (fig. S5, D and E). Accordingly, NR2F6 KO attenuated NR2F6 and RNA polymerase II binding and H3K27 acetylation at *Nacc1* R4 (Fig. 5E). Furthermore, OE of NR2F6 DNA binding mutant, but not NR2F6 WT, significantly reduced NACC1 expression by inhibiting the binding of endogenous NR2F6 to R4 region of *Nacc1* gene (fig. S5, F and G). Overall, these findings suggest that NR2F6 directly regulates *Nacc1* transcription but that its regulation of *Fkbp10* may be indirect.

Given these observations in mouse cells, we assessed NR2F6 function in regulating NACC1 and FKBP10 in human melanoma lines and patient-derived xenograft (PDX) tissues. We observed comparable NR2F6 expression in human melanoma tumor specimens, independent of genetic driver mutations (fig. S5, H to K), and NACC1 and FKBP10 were expressed in most human melanoma lines tested (fig. S5L). shRNA-based NR2F6 KD in human melanoma cell lines, A375 and Lu1205, decreased expression of NACC1 relative to controls, while FKBP10 was not affected by NR2F6 KD (fig. S5, M and N), suggesting that NACC1 regulation by NR2F6 is conserved in mouse and human melanoma.

**Fig. 6.**
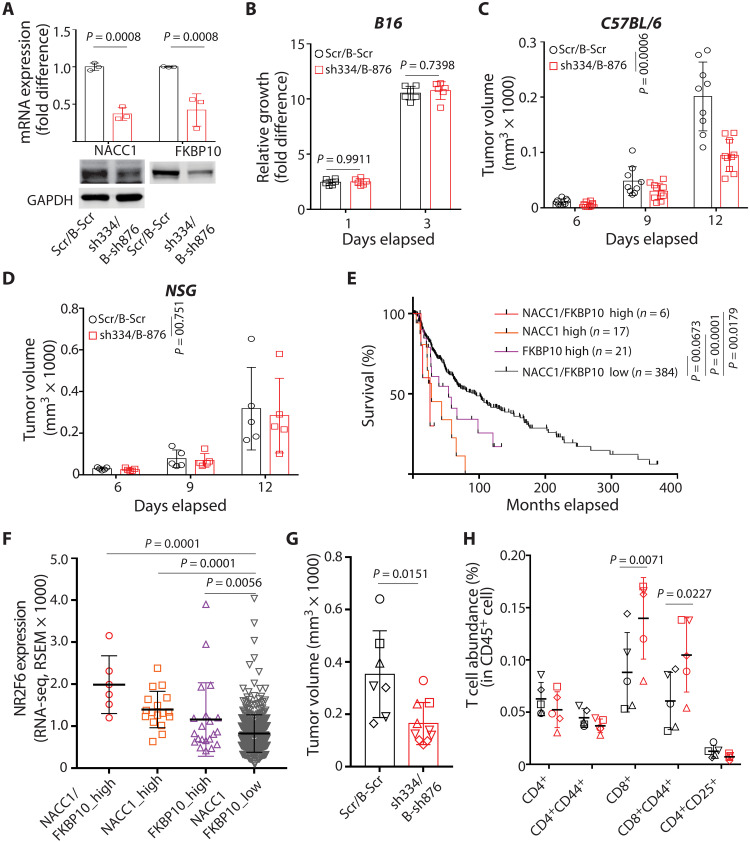
Loss of NACC1 and FKBP10 phenocopies NR2F6 loss. (**A**) B16F10 cells were transduced with scrambled shRNAs [Scr (puromycin resistant) and B-Scr (blasticidin resistant)] and one of two shRNAs (sh334 for NACC1 KD and B-sh876 for FKBP10 KD). mRNA and protein expression was assessed by qPCR and immunoblotting, respectively. *n* = 3 for each group. (**B**) Growth of transduced cells in vitro, as assessed by CellTiter-Glo. *n* = 6 for each group. (**C** and **D**) Transduced cells were used to inoculate C57BL/6 (C) or NSG (D) mice. Tumor growth was monitored at indicated time points. *n* = 9 and 10 mice for Scr/B-Scr and sh334/B-876 group, respectively. (**E**) Overall survival was assessed in patients with melanoma (SKCM in TCGA, *n* = 428) whose specimens showed both high NACC1 and high FKBP10 expression, high expression of just one, or low expression of both. (**F**) NR2F6 expression was assessed in groups of specimens in (E). (**G** and **H**) Scrambled (Scr/B-Scr) and NACC1/FKBP10 KD (sh334/B-sh876) B16F10 cells were engrafted into C57BL/6 mice. Tumors were collected 12 days later, and their volume was assessed (G). Different shapes of data points indicate the pooled cells for FACS analysis. *n* = 7 and 10 mice for Scr/B-Scr and sh334/B-876 group, respectively. (H) Shown is the abundance of T cell subtypes within all CD45^+^ cells based on FACS. *n* = 5 pooled samples for each group. Data are presented as means ± SD. Statistical significance was assessed by Student’s *t* test (G) with Holm-Sidak’s test (A and H), one-way ANOVA with Dunnett’s test (F), two-way ANOVA with Sidak’s test (B to D), or log-rank test (E).

To determine whether candidate NR2F6 effectors exhibit antitumor activity, we asked whether NACC1 and/or FKBP10 loss would phenocopy NR2F6 loss and attenuate tumor growth. To do so, we established B16F10 melanoma cells deficient in either NACC1 or FKBP10 (Fig. 5, F and I) and then injected them into immune-competent or immune-incompetent mice. In immune-competent mice, NACC1 or FKBP10 depletion attenuated tumor growth, 26.7% (sh336) to 37.6% (sh334) and 42.9% (sh876) to 46.8% (sh486), respectively (Fig. 5, G and J). These effects were not seen in immune-incompetent mice (Fig. 5, H and K) but resembled those seen in NR2F6-depleted melanoma. By contrast, in comparable analyses performed in cultured B16F10 NACC1 or B16F10 FKBP10 KD lines, we did not observe these growth changes (fig. S5, O and P), suggesting that NACC1 or FKBP10 loss suppresses tumor growth by augmenting antitumor immunity. Notably, the combined loss of NACC1 and FKBP10 further decreased tumor growth (by 52.8%) in immune-competent but not immune-compromised mice, again without altering cell growth in vitro (Fig. 6, A to D).

Notably, patients whose melanoma specimens showed high NACC1 and FKBP10 expression exhibited the worst overall survival, and those specimens also exhibited the highest NR2F6 expression relative to specimens expressing low NACC1 and FKBP10 (Fig. 6, E and F). Notably, we observed a robust increase in the number of infiltrated CD8^+^ T effector cells, but not other immune cells, in melanoma tumors doubly deficient in NACC1 and FKBP10, changes resembling those seen in NR2F6-ablated melanoma (Fig. 6, G and H, and fig. S5, Q to S). These observations strongly suggest that NACC1 and FKBP10 serve as NR2F6 effectors in mediating immune evasion in melanoma.

### Antitumor immunity seen following the loss of tumor-intrinsic NR2F6 expression is augmented by systemic NR2F6 loss

**Fig. 7.**
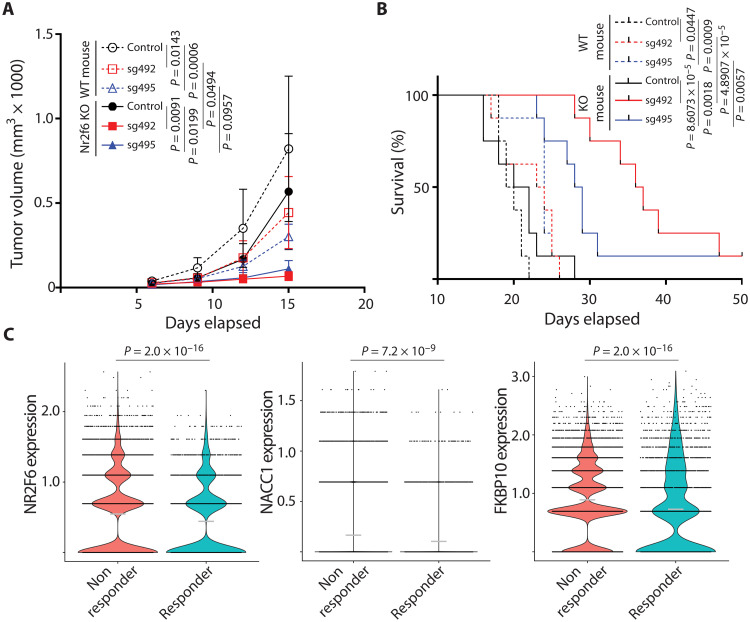
Combined tumor cell-intrinsic and cell-extrinsic NR2F6 loss synergize to inhibit melanoma growth. (**A** and **B**) B16F10 control (NR2F6 WT) or NR2F6 KO (sg492 and sg495) cells were used to inoculate C57BL/6 control (NR2F6 WT) or NR2F6 KO mice. Tumor volumes (A) and overall animal survival (B) were monitored at indicated time points. *n* = 8 mice for each group. (**C**) Violin plots compare the expression of NR2F6, NACC1, and FKBP10 in malignant melanoma cells from patients classified as ICT responders (*n* = 12) and nonresponders (*n* = 23). Each dot represents a single malignant cell, and gray horizontal lines are the mean expression in each group. Statistical significance was assessed by two-way ANOVA with Tukey’s test (A), log-rank test (B), or Wilcoxon test (C).

It was previously demonstrated that tumor-extrinsic NR2F6 expression could block antitumor immunity in genetically ablated NR2F6 KO mice. Those mice, which lack NR2F6 expression in stromal components, showed increased antitumor immunity relative to NR2F6 WT mice (*31*, *32*). Thus, we asked whether antitumor immunity seen following the loss of tumor-intrinsic NR2F6 expression would be augmented in mice genetically deficient in NR2F6. To do so, we inoculated both WT and NR2F6 global KO mice with either WT or NR2F6-deficient B16F10 cells. WT mice with NR2F6 depletion in tumor cells showed a 45.85 to 62.95% reduction in tumor growth, while those with systemic NR2F6 loss showed a 30.77% reduction (Fig. 7A). By contrast, NR2F6 depletion both in tumors and systemically promoted a further decrease in melanoma growth by 86.37 to 91.69% (Fig. 7A). In agreement, NR2F6 KO mice inoculated with NR2F6 KO tumor cells exhibited prolonged survival compared with NR2F6 WT mice inoculated with NR2F6 KO tumor cells or NR2F6 KO mice inoculated with NR2F6 WT tumor cells (Fig. 7B). These findings overall suggest that both tumor-intrinsic and tumor-extrinsic NR2F6 expression contribute to antitumor immunity in this context and determine the degree of tumor growth inhibition.

Important corroboration of our findings, tumor-intrinsic NR2F6, came from independent scRNA-seq analysis of human melanoma specimens: NR2F6, NACC1, and FKBP10 expression in malignant melanoma cells was significantly higher in samples from patients that are nonresponsive to anti–PD-1–based ICT (Fig. 7C). Given the correlation in melanoma specimens, we set to correlate NR2F6, NACC1, and FKBP10 expression with patients’ overall survival and response to ICT in other cancer types. scRNA-seq analysis of nonmetastatic primary breast tumors from patients subjected to a single dose of anti–PD-1 ICT revealed higher NR2F6 and FKBP10 expression in nonresponders [fig. S7A; denoted “nonexpanders” (NE)]. In this patient subset, NACC1 expression did not correlate with responses to ICT (fig. S7D) (*45*). Analysis of bulk RNA-seq data from the primary tumors of 224 pancreatic cancer patients showed that NR2F6 expression was highly correlated with NACC1 and FKBP10 expression (fig. S7, B and C). Within the same cohort, NACC1 and FKBP10 expression levels showed a significant inverse correlation with patient survival (fig. S7C), while NR2F6 expression did not (fig. S7C). In 64 non–small cell lung cancer specimens, NR2F6, NACC1, and FKBP10 expression was higher in tumors than in the adjacent normal tissues (fig. S7E), and high expression of these genes was correlated with poor survival of patients (fig. S7F). As in melanoma, NR2F6 expression was highly correlated with NACC1 or FKBP10 expression (fig. S7G). These observations points to a tissue-specific variation in the expression of NR2F6 and its effectors, NACC1, and FKBP10, in different cancer types. The latter is expected to influence NR2F6-driven tumor-intrinsic changes that determine tumor immune evasion capabilities.

## DISCUSSION

Deregulation of NR expression reportedly alters the tumor response to antitumor immunity. Here, we identify a previously undisclosed role for the orphan nuclear receptor NR2F6 in tumor-intrinsic control of immune evasion by melanoma cells, a function highly relevant to ICT effectiveness. NR2F6 expression in tumor cells was inversely correlated with that of IFN-γ signature genes and with overall melanoma patient survival, observations that led us to focus on NR2F6 among >40 candidate NRs. Our findings establish a tumor-intrinsic function for NR2F6 and identify NACC1 and FKBP10 as their downstream effectors in inhibiting antitumor immunity. *Nacc1* gene expression is likely regulated directly by NR2F6 binding to an intronic region, while FKBP10 regulation by NR2F6 may be indirect.

Inhibition of NR2F6 expression by either shRNA KD or CRISPR-mediated KO inhibited melanoma growth in immune-competent but not immune-incompetent mice, an outcome phenocopied by NACC1 and FKBP10 loss. Notably, analysis of melanoma patient data provided important support for our findings, revealing higher expression of NR2F6/NACC1/FKBP10 in nonresponders to ICT. Inhibition of NACC1 and FKBP10 in tumor cells enhances CD8^+^ T cell recruitment to limit tumor growth by a number of possibilities. Among them is the notion that NACC1 (aka NAC1) protein may serve as a scaffold between MAVS and TBK1 proteins, implicated in antiviral signaling, which could activate innate immunity (*46*). As a determinant of regulatory T (T_reg_) cell activity, NACC1 can also destabilize T_reg_, resulting in suppression of tolerance (*47*). Notably, NACC1 KO in B16-OVA tumors reportedly augments CD8 T cell infiltration by decreasing Lactate Dehydrogenase (LDHA) expression (*48*), which changes the TME. While this finding is consistent with the activation of antitumor immunity by NACC1 KD reported here, further studies are required to determine how NACC1 promotes tumor immune evasion depending on cell and tumor types. While a role for FKBP10 in regulating tumor immunity has not been reported, FKBP10 functions in collagen biogenesis, which likely affects TIL function, suggesting that remodeling of the extracellular matrix after FKBP10 down-regulation in NR2F6 KO melanoma may govern the degree and effectiveness of antitumor immunity (*49*–*51*).

NR2F6 and NACC1 function as transcriptional activators or repressors of target genes by forming homo- or heterodimers with another transcriptional regulator (*29*, *52*). In addition to a dimerization partner, a transcriptional coregulator often determines whether the target genes will be activated or repressed, which explains the possible disconnect between loss- and gain-of-function studies. The OE of NR2F6 WT did not promote tumor growth, while NR2F6 DNA binding mutant (MT) attenuated tumor growth. Along these lines, NR2F6 MT, but not WT, bound to the *Nacc1* gene and regulated *Nacc1* transcription. Likewise, OE of NACC1 did not rescue growth inhibition seen upon NR2F6 loss, suggesting that (i) additional genes/pathways may be required for rescued growth or (ii) NR2F6 loss cause irreversible changes (e.g., genome accessibility) that could not be rescued by NACC1 and FKBP10 expression. Further studies will be required to dissect the mechanisms underlying the control of NR2F6/NACC1 pathway components. Unlike NACC1, FKBP10 expression was not affected by NR2F6 loss in human melanoma cells. In agreement, NR2F6 expression did not correlate with FKBP10 expression in melanoma cells [quantitative reverse transcription polymerase chain reaction (qRT-PCR) and cancer cell line encyclopedia (CCLE)]. In contrast, FKBP10 expression was significantly correlated with NR2F6 expression in specimens from patients with melanoma, pancreatic, and lung cancer, suggesting the requirement of TME components for the NR2F6 control of tumor-intrinsic FKBP10 expression.

Of interest is the observation that while NR2F6 KD effectively induced immune cell infiltration, primarily by CD8^+^ T cells, which limited tumor growth, NR2F6 OE did not enhance tumor growth, suggesting either that additional cofactors are needed for NR2F6 function or that tumor-intrinsic NR2F6 activity depends on the availability of tumor-extrinsic factors. The latter is also plausible, given that a tumor-extrinsic role for NR2F6 in controlling antitumor immunity in two distinct autochthonous tumor models was reported in mice globally deficient in NR2F6 (*31*, *32*). Previous studies of graft models using subcutaneous injection of NR2F6-expressing WT tumor cells suggest that NR2F6 regulates immune system activities independently of its tumor cell expression (*32*). These findings suggest that NR2F6 has a function in the TME and the CD8^+^ T cells, as evidenced by the antitumor effect of acute lymphatic NR2F6 silencing before their adoptive transfer (*33*).

As noted, it was previously reported that NR2F6 global KO mice harboring mouse melanoma and colon cancer models showed enhanced responses to programmed death-ligand 1/PD-1 ICT relative to comparable WT mice (*32*). Here, we report that combined ablation of NR2F6 both globally and in the injected melanoma cells synergistically antagonized tumor growth in mice, suggesting that distinct albeit complementing activities enhance antitumor immunity. Future studies should address potential tumor-extrinsic factors that regulate the degree of antitumor immunity. Among candidates are nonimmune stromal components, which express higher NR2F6 levels than do immune cell subtypes (fig. S1, B and C). The latter finding is of interest given the role reported for stroma in controlling immune cell infiltration and activation (*53*, *54*). NR2F6 may control stromal cell activities in a way that affects the recruitment or activation of CD8^+^ T cells and promotes their ability to infiltrate tumors.

Could NR2F6 serve as a target for therapy? Such a strategy has been suggested by the Baier group who demonstrated the importance of NR2F6 as an intracellular immune checkpoint in effector T cells (*55*) and later showed that mice genetically deficient in NR2F6 and then inoculated with tumor cells exhibit relatively decreased tumor growth (*31*, *32*). Here, we add an important component to this equation by demonstrating that tumor-intrinsic NR2F6 expression is equally relevant and that combining tumor-intrinsic and -extrinsic NR2F6 ablation synergizes to block tumor growth. This finding justifies the development of NR2F6 inhibitors that could be administered systemically to target both tumors and the TME. Initial efforts to identify these compounds have been reported (*56*) and should gain more traction, given our findings reported here.

## MATERIALS AND METHODS

### Experimental models

All studies conducted in mice were approved by the Institutional Animal Care and Use Committee of Sanford Burnham Prebys Medical Discovery Institute (AUF#21-032). Murine melanoma (B16F10 and YUMM1.7), breast (4T-1), lung (LLC), and pancreatic ductal adenocarcinoma (KPC) cancer cells were injected subcutaneously (2.0 × 10^5^ B16F10 or 1.0 × 10^6^ YUMM1.7 cells; 1.0 × 10^6^ 4T-1 cells; 1.5 × 10^6^ LLC cells) into the lower right flank of 6- to 8-week-old male C57BL/6 (B16F10, YUMM1.7, YUMMER1.7, and LLC), female Balb/C (4T-1), NSG (B16F10 and YUMM1.7), or NR2F6 KO (*32*) mice. Tumor growth was monitored weekly using calipers. At indicated time points, tumors were collected, weighed, and assessed for immune phenotypes using flow cytometry. To assess the tumor response to immune checkpoint antibodies, mice were grafted with B16F10 (2.0 × 10^5^ cells, s.c.) cells and treated with 200 μg per mouse anti-CD279 (PD-1) [RMP1-14 (BE0146, Bio X Cell)]. Antibodies were injected (intraperitoneally) three to five times (every 3 days starting at indicated dates). To assess the percent survival of animals, mice bearing tumors exceeding 2000 mm^3^ were defined as “dead.”

### Analysis of cancer gene expression and patients’ clinical outcomes

To correlate the expression of 48 NRs with IFN-γ-signature genes (*16*), mRNA expression data (*z* scores relative to diploid samples) of NR genes in The Cancer Genome Atlas datasets (bulk RNA-seq) were downloaded from cBioPortal. Spearman’s correlation coefficients assessing the expression of NRs and IFN-γ signature genes were calculated. A corresponding heatmap based on correlation coefficients and *P* value data was plotted using Prism 7.0 or 9.0 software (GraphPad).

The IFN-γ index for each patient sample was calculated as an average rank percentile of 10 IFN-γ signature genes in a total of 443 samples. On the basis of those values, groups of patients with an index of >75% or <25% were selected for further analysis of correlation with overall survival, which was obtained from cBioPortal. The correlation of IFN-γ index with patient’s survival was assessed by Kaplan-Meier survival analysis using Prism 7.0 or 9.0 (GraphPad), as was the correlation of patient overall survival with NR expression. Briefly, two groups of patients, those in the 25th and 75th quartiles of NR expression, were compared by Kaplan-Meier survival analysis. NRs with a significant *P* value in the log-rank (Mantel-Cox) test were plotted in the heatmap as associated with either favorable or unfavorable overall survival.

Patient response to ICT and corresponding NR expression in malignant cells was plotted using published data and analyses (Single Cell Portal, singlecell.broadinstitute.org/single_cell). To assess NR expression in each cell type in melanoma tumors, we downloaded scRNA-seq data [GSE72056; (*57*)] and calculated the percent expression of each NR in different cell types by dividing the number of cells expressing that NR by the total cell count.

The RNA-seq data of human melanoma cell lines (CCLE; Cancer Cell Line Encyclopedia) were downloaded from DepMap (www.depmap.org) and used to assess NR2F6, NACC1, and FKBP10 expression. The expression data (RNA-seq) of NR2F6, NACC1, and FKBP10 in human PDX tissues were provided by M.H. (The Wistar Institute). The bulk RNA-seq data from 224 primary tumors of patients with pancreatic cancer and corresponding survival data (https://doi.org/10.1101/2022.05.04.490552) were provided by R.S. (Oregon Health Science University). The scRNA-seq data of human breast cancer (nonmetastatic primary invasive carcinoma) from 29 patients treated with one dose of anti–PD-1 ICT approximately 9 ± 2 days before surgery was previously reported (*45*).

### RNA-seq analysis

RNA samples were prepared from bulk tumors, MACS-sorted tumor cells, and cultured cells for gene expression analysis. To purify tumor cells from bulk tumor tissue, collected B16F10 tumors were minced, chopped, and incubated in collagenase D solution [0.1% (w/v) collagenase D, 0.5% (w/v) bovine serum albumin (BSA), and deoxyribonuclease (DNase) (100 μg/ml) in phosphate-buffered saline (PBS)] for 1 hour at 30°C. A single-cell suspension was obtained using a cell strainer (70 μm; Falcon). Tumor cells were purified by depletion of stromal cells using a Tumor Cell Isolation kit (Miltenyi Biotech). The purity of isolated tumor cells was validated by fluorescence-activated cell sorter (FACS) using a CD45 antibody. RNA from bulk tumors, MACS-sorted tumor cells, and cultured cells were purified using a GenElute total RNA purification kit (Sigma-Aldrich).

For library construction, polyadenylate [poly(A)] RNA was isolated using NEBNext Poly(A) mRNA Magnetic Isolation Module, and bar-coded libraries were constructed using the NEBNext Ultra Directional RNA Library Prep Kit for Illumina (NEB, Ipswich, MA). Libraries were pooled and sequenced from a single end (1X75) on an Illumina NextSeq 500 system using the High Output V2 Kit (Illumina Inc., San Diego, CA) at a sequencing depth of 23 to 31 million reads. Raw reads were trimmed to remove Illumina TruSeq adapters and poly(A)/poly(T) sequences using Cutadapt version 2 (*58*). Reads were then aligned to mouse genome version mm10 and Ensembl gene annotations version 84 using STAR version 2.7.0d_0221 and alignment parameters from ENCODE long RNA-seq pipeline (https://github.com/ENCODE-DCC/long-rna-seq-pipeline). We obtained gene level estimated counts and transcripts per million using RSEM version 1.3.1. FastQC version 0.11.5 (www.bioinformatics.babraham.ac.uk/projects/fastqc/) and MultiQC version 1.8 were used to assess the quality of trimmed reads and alignment to genome/transcriptome. Genes expressed at low levels were removed from downstream analysis by selecting those with RSEM estimated counts equal to or greater than five times the total number of samples. Differential expression comparisons were performed using the Wald test implemented in DESeq2 version 1.22.2. Genes with Benjamini-Hochberg corrected *P* value < 0.05 and fold change ≥ 1.5 or ≤ −1.5 were identified as differentially expressed. Pathway analysis was performed using Ingenuity Pathway Analysis (QIAGEN, Redwood City, USA).

The scRNA-seq dataset of human metastatic melanoma samples was analyzed as outlined [(*59*); www.biorxiv.org/content/10.1101/2022.08.11.502598v1]. NR2F6, NACC1, and FKBP10 expression was compared between anti–PD-1 therapy responding (*n* = 12) and nonresponding (*n* = 23) patients using the Wilcoxon signed rank test. Samples acquired from patients either before or after treatment were grouped.

A total of 64 non–small cell lung cancer cases stage 1A or 1B (tumor and normal adjacent tissue) were analyzed for NR2F6 and its downstream effectors NACC1 and FKBP10 gene expression. The diagnosis of lung cancer was pathologically determined. A pathologist performed tumor staging using the seventh edition of the *AJCC’s Cancer Staging Manual*. Written informed consent or waiver of consent was obtained for experimentation with all the enrolled participants. This study was approved by the appropriate Institutional Review Boards and conducted in accordance with the Declaration of Helsinki. All total RNA-seq samples were pooled and sequenced on NovaSeq 6000 S2 using Illumina Stranded Total. The gene expression quantification analysis was performed for all samples using STAR/RSEM tools. Expression counts were transformed to log_2_ CPM (counts per million reads) for analysis. Scatter plots and box plots were created using the ggscatter and ggboxplot functions respectively from the ggpubr package in R. Kaplan-Meier plots were generated using the survival (v3.5-5) and survminer (v0.4.9) package with ggplot2 (v3.4.1). All statistical tests were implemented in R (v4.2.2) and RStudio (v2022.12.0+353).

### Plasmid construction and mutagenesis

DNA plasmids were constructed using the pLX302 and pLX304 Gateway system (Addgene, #25890). Briefly, PCR-amplified mouse *Nr2f6*, *Nr1h3*, and *Nr3c1* cDNAs were cloned into the lentiviral Gateway Vector using LR clonase II and a pENTR-D-TOPO cloning kit (Thermo Fisher Scientific). Mutant Nr2f6 (C112S) incapable of binding DNA was generated by introducing point mutations into the pENTR-Nr2f6 construct using the QuikChange II XL Site-Directed Mutagenesis Kit (Agilent). Inserts were subsequently cloned into pLX304 (lentiviral) expression plasmids. shRNA clones harboring a blasticidin resistance gene were generated by cloning validated oligonucleotides into the Eco RI/Age I sites of pLKO.1-Blast (Addgene, #26655). Gene-specific shRNA lentiviral vectors with a pLKO.1 backbone were purchased from Sigma-Aldrich.

For NACC1 and FKBP10 OE, mouse *Nacc1* was cloned into pLX304 expression plasmids using the Gateway Cloning system. Briefly, PCR-amplified mouse *Nacc1* cDNA was cloned into pENTR-D-TOPO, which was subsequently cloned into pLX304. For *Fkbp10*, PCR-amplified mouse *Fkbp10* cDNA was cloned into pLV lentiviral plasmids using Eco RI/Xho I sites.

### Production and infection of viral particles

Lentiviral particles were prepared using standard protocols. Briefly, HEK293T cells were transfected with lentiviral plasmid and the second-generation packaging plasmids psPAX2 (Addgene, #12260) and pMD2.G (Addgene, #12259) using CalFectin (SignaGen) or JetPrime (Polyplus). Viral supernatants were collected 48 hours later, filtered using a syringe filter (0.45-μm pore size), and concentrated by centrifugation (13,000*g* for 2 hours). Titrated viral particles and polybrene (8 μg/ml; Sigma-Aldrich) were applied to cultures (2 × 10^5^ cells per well in six-well plates) and subjected to spinoculation (1500*g* for 30 min) to infect melanoma cells. The efficiently infected cells were selected in cultures containing either puromycin (1 to 1.5 μg/ml; InvivoGen) or blasticidin (5 to 10 μg/ml; InvivoGen), as appropriate.

### Gene silencing

To knock down genes, pLKO.1clones for respective genes were purchased (Sigma-Aldrich). pLKO.1 clones for each gene were as follows: Nr2f6 (TRCN0000026194, TRCN0000026226, TRCN0000033660, TRCN0000033661, TRCN0000033662, and TRCN0000033663), Nacc1 (TRCN0000071334 and TRCN0000071336), and Fkbp10 (TRCN0000339486 and TRCN0000111876). For double KDs, oligonucleotides of the same sequence (TRCN0000111876) were cloned into pLKO.1-blast (Addgene, #26655). Cells transduced were selected in culture containing puromycin (InvivoGen) or blasticidin (Gibco).

To knock out Nr2f6 using CRISPR, Nr2f6-specific sgRNAs (Thermo Fisher Scientific, CRISPR553495_SGM and CRISPR553492_SGM) were labeled with Cy3 using a Label IT kit (Mirus). B16F10 cells were transfected with labeled sgRNAs and Cas9 protein (Thermo Fisher Scientific) using CRISPRMax Cas9 transfection reagent (Thermo Fisher Scientific). Cells were collected after 24 hours of culture and subjected to FACS sorting to isolate Cy3^+^ cells. Nr2f6 KO was validated by immunoblotting and subsequent sequencing of regions targeted by sgRNAs.

### Assessment of cell growth in culture

The growth of cultured cells was measured by assessing adenosine 5′-triphosphate content of viable cells using CellTiter-Glo (Promega). Briefly, 2000 to 2500 cells were placed into 96-well plates with clear bottoms (Nunc), CellTiter working solution was added to wells, and luminescence was measured with a CLARIOstar microplate reader. Luminescence at days 1 and 3 was calculated as a fold difference relative to luminescence at day 0.

### Immunoblotting

Immunoblotting samples were processed using standard protocols with slight modifications. Briefly, cells were lysed by incubation in radioimmunoprecipitation assay (RIPA) buffer with 0.1% SDS [50 mM tris-HCl (pH 7.4), 1% (v/v) NP-40, 0.1% (w/v) sodium deoxycholate, 0.1% (w/v) SDS, 150 mM NaCl, 1 mM EDTA, and a protease/phosphatase inhibitor cocktail (Thermo Fisher Scientific)] and three freeze-thaw cycles. Tumor tissues were lysed by incubation in RIPA buffer with 0.5% SDS [50 mM tris-HCl (pH 7.4), 1% (v/v) NP-40, 0.1% (w/v) sodium deoxycholate, 0.5% (w/v) SDS, 150 mM NaCl, 1 mM EDTA, and a protease/phosphatase inhibitor cocktail (Thermo Fisher Scientific)] followed by homogenization using a Tissuemiser (Thermo Fisher Scientific). Lysates were boiled in Laemmli buffer before separation on SDS–polyacrylamide gel electrophoresis and then transferred to a polyvinylidene difluoride membrane. Membranes were incubated with blocking solution [TBS (tris-buffered saline); 10 mM tris-HCl (pH 8.0) and 150 mM NaCl)] containing 0.1% Tween 20 and 5% nonfat milk followed by incubation with appropriate primary antibodies overnight at 4°C. Membranes were washed with TBS and incubated 1 hour at room temperature with secondary antibody [Alexa Fluor 680–conjugated goat anti-rabbit, goat anti-mouse, and donkey anti-goat (Life Technologies) or IRDye 800–conjugated goat anti-mouse (Rockland Immunochemicals)] or horseradish peroxidase (HRP)–conjugated anti-mouse or anti-rabbit IgG antibodies (Cell Signaling Technology). Bands on blots incubated with fluorescent antibodies were visualized and quantified using an Odyssey Infrared Imaging System (LiCoR Biosciences). Bands with HRP activity were visualized using a ChemiDoc Imaging system (Bio-Rad) after incubating blots with West Pico plus Chemiluminescent substrate (Thermo Fisher Scientific) or Immobilon Forte Western HRP substrate (Millipore). The following antibodies were used to detect indicated proteins: NR2F6 (60117-1-Ig and 60117-2-Ig, Proteintech), NAC1 (#4183 and #4420, Cell Signaling Technology), FKBP10 (12172-AP, Proteintech), and V5-tag (7/4, BioLegend).

### Immunofluorescent

Paraffin tumor sections were prepared following routine protocols for immunohistochemistry. Sections were stained with anti-CD8α (1:500 dilution; Abcam, EPR21769) and then with the secondary antibody conjugated with Alexa Fluor 568 (1:500 dilution; Invitrogen). Nuclei were stained with 4′,6-diamidino-2-phenylindole (DAPI) (Vector Laboratory). Immunofluorescence-stained slides were visualized with a fluorescent microscope aided by Slidebook software (Olympus). The number of CD8^+^ cells was counted in randomly picked four fields of each slide. The number of CD8^+^ staining was directly counted or quantified using the particle analysis tool of ImageJ software (National Institutes of Health). The percent abundance of CD8^+^ staining was calculated by normalizing CD8^+^ count to the DAPI^+^ count.

### RNA extraction and qPCR

Total RNA was purified from cells with GenElute (Sigma-Aldrich), a PureLink RNA kit (Invitrogen), or a Quick-RNA kit (Zymo Research). Purified RNA was reverse-transcribed using a high-capacity cDNA synthesis kit (Applied Biosystems). qPCR was carried out with a CFX Connect Real-Time PCR Detection System (Bio-Rad) using SsoAdvanced Universal SYBR Green Supermix (Bio-Rad). The sequences of primers used were as follows: 18*S* ribosomal RNA (internal control for normalization (5′-GTAACCCGTTGAACCCCATT-3′; 5′-CCATCCAATCGGTAGTAGCG-3′), mouse Nr2f6 (5′-GAGGACGATTCGGCGTCAC-3′; 5′-GTAATGCTTTCCACTGGACTTGT-3′), human NR2F6 (5′-GAGCGGCAAGCATTACGGT-3′; 5′-GGCAGGTGTAGCTGAGGTT-3′), mouse Nacc1 (5′-GCGGCTACAGGGACTATACTG-3′; 5′-CCGGAAGTAAGAGCTACTAGCG-3′), Human NACC1 (5′-CTGGCTCCTACCACAATGAGG-3′, 5′-TGGCCGACGTTCATCATGC-3′), mouse Fkbp10 (5′-TACTGCCGTTGCTGTTGCTT-3′; 5′-GGGATGTGGTATCTCTCGATGAC-3′), Human FKBP10 (5′-TACAGTAAGGGCGGCACTTAT-3′; 5′-GAGGACGTGAAAGACCAGCG-3′), and mouse Cxcl10 (5′-CCAAGTGCTGCCGTCATTTTC-3′; 5′-GGCTCGCAGGGATGATTTCAA-3).

### Immune phenotyping of tumors using flow cytometry

To assess immune phenotypes of TILs, B16F10 tumors were collected at indicated times and then chopped and incubated in collagenase D solution [0.1% (w/v) collagenase D, 0.5% (w/v) BSA, and DNase (100 μg/ml) in PBS] for 1 hour at 30°C. A single-cell suspension was obtained using a cell strainer (70 μm; Falcon). Total cells were counted and a fraction (2 × 10^6^) of cells in FACS staining buffer [PBS (pH 7.4) containing 1% fetal bovine serum] was treated with the following sets of antibodies (1:200 dilution): cocktail 1 {CD45.2 (AF700), CD8 [allophycocyanin (APC)], CD4 (BV605), CD44 (APC/Cy7), CD25 [fluorescein isothiocyanate (FITC)], and purified CD16/32}, cocktail 2 {CD45.2 (AF700), MHCII (PB), CD11C (APC), CD11b (APC/Cy7), GR1 [phycoerythrin (PE)], F4/80 (FITC), NK1.1 (BV605), B220 (PE/Cy7), and purified CD16/32}, cocktail 3 [CD45.2 (AF700), CD8 (FITC), CD4 (BV605), CTLA4 (Peridinin-Chlorophyll-Protein/Cy5.5), LAG3 (PE), PD-1 (APC), and TIM-3 (PE/Cy7)], cocktail 4 [CD45.2 (AF700), CD8 (BV421), PD-1 (APC), and TIM-3 (PE/Cy7)], cocktail 5 [CD45.2 (AF700), CD11c (APC), MHCII (PB), CD8a (BV421), XCR-1 (PE), CD4 (BV605), and CD11b (APC/Cy7)], cocktail 6 {CD45.2 [(AF700), CD11b (APC/Cy7), Gr1 (FITC), Ly6C (PE/Cy7), Ly6G (PE), and F4/80 (APC/Cy7)]}, and cocktail 7 {CD45.2 [(AF700), CD11b (APC/Cy7), F4/80 (FITC), CD38 (BV421), Ly6C (PE/Cy7), and Ly6G (PE)]} for 20 min at 4°C. All antibodies were from BioLegend, except TCF1 (BD Biosciences). Stained cells were fixed in 1% formaldehyde (Sigma-Aldrich) in PBS (pH 7.4) for 15 min at 4°C and analyzed with BD LSRFortessa (BD Biosciences) flow cytometry. To stain intracellular and nuclear markers, cells were fixed with Foxp3 fix/permeabilization kit (eBioscience) and then treated with the following set of antibodies [TCF1 (FITC), EGR2(APC), or CD206 (BV605)]. To assess intracellular cytokines in infiltrated CD4^+^ and CD8^+^ cells, a fraction (2 × 10^6^) of cells prepared from tumors were stimulated with phorbol 12-myristate 13-acetate (10 ng/ml)/ionomycin (0.5 μg/ml)/brefeldin A (1 μg/ml) for 16 hours. Cells were stained with a cocktail of antibodies for surface markers [CD45.2 (AF700), CD4 (BV605), and CD8 (APC)] followed by staining with intracellular cytokine antibodies [IFN-γ (APC), TNFα (FITC), and IL-2(PE)]. Cell type abundance was calculated as a percentage of CD45^+^ cells.

### CD8 T cell depletion

Mice were treated with anti-CD8^+^ antibody {2.43 (BE0061, Bio X Cell) or control IgG (200 μg per mouse) [rat IgG2b (BE0090, Bio X Cell)]}. Antibodies were injected (intraperitoneally) every 3 days starting 1 day before tumor cell inoculation. Depletion efficiency was assessed using flow cytometry of blood samples collected on day 8 after tumor inoculation.

### Chromatin immunoprecipitation qPCR

Putative regulatory regions for NR2F6 binding on the *Nacc1* and *Fkbp10* genes were predicted using ReMap2022 (remap2022.univ-amu.fr). Specific primers for each region were designed and assessed by qPCR following validation for amplicon size using agarose gels.

For ChIP analysis, protein-crosslinked DNA fragments were immunoprecipitated using the Magna ChIP Kit (Millipore). For immunoprecipitation, ChIP-validated antibodies were used for NR2F6 (*60*) (H9929A, Perseus Proteomics), RNA polymerase II (Rbp1, 4H8, Cell Signaling Technology), and acetyl-H3K27 (D5E4, Cell Signaling Technology). Purified DNA fragments were then assessed by qPCR using region-specific primers. The relative abundance to input (5% of preimmunoprecipitation) was calculated. The sequence of primers used was as follows: R1-1 for Nacc1 (5′-GGGGAGCAGGTTAGGAAAAC-3′; 5’-AGTTGGCCAAGGTGTCAGAG-3′), R1-2 for Nacc1 (5′-TTTTGCTTTGCCTGGACTTT-3′; 5′-CCCTTCCAAAACAAGGAACA-3′), R2 for Nacc1 (5′-AGGTGTATTGCCTGGACTGC-3′; 5′-CAAAACCCCAACCAATCATC-3′), R3 for Nacc1 (5′-GGGCCACAGACTGTCGTATT-3′; 5′-GATCAGTGCTGGGGGATAGA-3′), R4 for Nacc1 (5′-GGCAAGAAGACCAAGACTCG -3′; 5′-CCTCACAGCCATGCCTTTAT-3′), R1-1 for Fkbp10 (5′-GCAAAGTGGACGAAGTCACA-3′; 5′-CGGGGTAACAAGAGGTGTGT-3′), R1-2 for FKBP10 (5′-CCGTTGCTGTTGCTTCTACA-3′; 5′-CTGGAAGCTCAGGAAAGTGG-3′), R2-1 for FKBP10 (5′-TGCTTATCTGTCCCGTTTCC-3′; 5′-GACACACATGCCCATGAGAC-3), and R2-2 for FKBP10 (5′-AGACTGAGGGGCACTCTGAA-3′; 5′-GGACAGCAATCTCAGGGGTA-3).

### Statistical analysis

Statistical analyses were performed using Prism software (version 7.00, GraphPad). For the comparison of the means of two groups with normal (or approximately normal) distributions, an unpaired *t* test was applied. In multiple *t* tests between two groups, adjusted *P* values were computed using the Holm-Sidak method. The statistical significance of gene expression levels in two cell populations based on scRNA-seq data was determined by Wilcoxon-signed rank test. To compare means between >2 groups, we used one-way analysis of variance (ANOVA) with multiple comparison corrections (Dunnett’s test). For animal experiments, we used two-way ANOVA (time and treatment) with Dunnett’s, Tukey’s, or Sidak’s multiple comparison test. For Kaplan-Meier plots to compare overall survival, we used a log-rank (Mantel-Cox) test to determine the significance of differences between groups.

## Notes

View/request a protocol for this paper from *Bio-protocol*.
