# Supplementary material for: Melanoma-intrinsic NR2F6 activity regulates antitumor immunity
Source: Sci Adv. 2023 Jul 5;9(27):eadf6621. doi: 10.1126/sciadv.adf6621 (PMC10321753; doi:10.1126/sciadv.adf6621)
Supplement: Supplementary file 1 — Figs. S1 to S6 [file sciadv.adf6621_sm.pdf]

Science Advances
AAAS

Supplementary Materials for

Melanoma-intrinsic NR2F6 activity regulates antitumor immunity

Hyungsoo Kim *et al.*

Corresponding author: Ze'ev A. Ronai, zeev@ronailab.net; Hyungsoo Kim hkim@sbpdiscovery.org

Sci. Adv. **9**, eadf6621 (2023)
DOI: 10.1126/sciadv.adf6621

This PDF file includes:

Figs. S1 to S6

Legend for Supplemental Figures

Supplementary figure S1.

Identification of NRs associated with anti-tumor immunity. (A) Overall survival of melanoma patients with a high (> 75th percentile) or low (<25th percentile) -IFNγ signature index (see Methods), based on Kaplan-Meier analysis. (B) Average (TPM) and percent expression of 48 NRs in indicated cell types, based on scRNAseq of human melanoma tissues (*57*). (C) Average (TPM) and percent expression of NR2F6 in indicated cell types, as assessed using the same dataset.

Supplementary figure S2.

Validation of control anti-tumor immunity by candidate NRs. (A) NR2F6 expression was assessed in indicated mouse melanoma, colon (MC38), breast (4T-1), and pancreatic (KPC) cancer lines by qPCR. n=3 for each group. (B) B16F10 cells stably expressing V5-tagged NR1H3 or NR3C1 were established by transduction with the corresponding lentivirus constructs. Cell lysates were analyzed by immunoblotting with a V5-tag antibody. (C) Growth of cultured cells established in (B), as assessed *in vitro* using CellTiter-Glo. Relative fold-difference in luminescence values on days 1 and 3 were calculated relative to luminescence at day 0. n=6 for each group. (D) DepMap (https://depmap.org/portal/) scores indicate effects on cell growth following KO of indicated NRs in human melanoma cell lines. Each data point indicates a different line. (E) C57BL/6 mice were inoculated with cells and treated with anti-PD-1 antibody (RMP1-14) on days 6, 9, 12, and 15 (arrows). Tumor volumes were monitored at indicated time points. n=mice for each group. (F-G) Cells established in (Figure 2A) were engrafted into C57BL6 (F) or NSG (G) mice, and tumor volumes were monitored at indicated time points. n=9 mice (F) for each group (F). n=11, 5 and 6 mice for Scr, sh194 and sh226 in (G). (H) Growth of control and CRISPR-KO B16F10 cells (Figure 2D), as assessed using CellTiter-Glo. n=6 for each group. (I) Lysates established from mouse tumors (Figure 2E) were analyzed by immunoblotting for indicated proteins. (J) The growth of cultured YUMM1.7 cells described in (Figure 2G) was assessed *in vitro* using CellTiter-Glo. n=6 for each group. (K) B16F10 cells stably overexpressing either WT NR2F6 or the C112S DNA-binding mutant were established by transduction of corresponding lentiviruses, followed

by immunoblotting of cell lysates for indicated proteins. (L) Growth of transduced cells *in vitro,* as assessed using CellTiter-Glo, as outlined in (C). n=6 for each group. (M-N) Transduced cells were used to inoculate C57BL/6 mice, and then tumor volumes (M, n=9 mice for each group) and mouse survival (N, n=8 mice for each group) were monitored at indicated time points. Data are presented as means ± SD. Two-way ANOVA assessed statistical significance with Dunnett's test (C, E, F, G, H, J, L, M), or long-rank test (N).

Supplementary figure S3.

Immune cell abundance in NR2F6 KO tumors. (A) Representative plots show the gating strategy used to acquire data in Figure 3C. (B) As in (Figure 3D-E), CD8 T cell abundance in Control (B-Scr) and NR2F6 KD (B-sh194 and B-sh226) YUMM1.7 tumors (4 tumors per group) was assessed by immunofluorescent staining with anti-CD8 antibody. Tumors were collected 10 days after inoculation in C57BL/6. CD8 staining was visualized, and the percent abundance of $CD8^+$ staining in $DAPI^+$ staining is shown. Scale bar (50 uM). n=4 mice (2 sections/mouse) for each group. (C) The abundance of T cells producing indicated cytokines in $CD45^+$ cells, as assessed by FACS. (D) The abundance of exhausted T cells in $CD45^+$ cells, as assessed by FACS. (E) The abundance of immune cell types in $CD45^+$ cells, as assessed by FACS using indicated surface markers [B cells ($B220^+$), macrophages ($CD11B^+F4/80^+$), MDSCs ($CD11b^+Gr1^+$), dendritic cells ($CD11c^+MHCII^+$), and NK cells ($NK1.1^+$)]. (F) Representative plots show the gating strategy used to acquire data in Figures 3F, S4G-I. (G) The abundance of M1 type ($CD38^+EGR2^-$) and M2 ($CD38^-EGR\text{-}2^+$ or $CD38^-CD206^+$) TAMs, as assessed by FACS. (H) The abundance of cDC1s ($CD8a^+XCR1^+$) and cDC2s ($CD8a^-CD11b^+$), as assessed by FACS. (I) The abundance of M-MDSCs ($CD11b^+Gr1^+Ly6C^+Ly6G^-$), as assessed by FACS. n=4 mice for each group (C to E, G to I). Data are presented as means ± SD. Statistical significance was assessed by one-way ANOVA with Dunnett's test (B, C, D, E, G, H, I).

Supplementary figure S4.

Identification of putative NR2F6 effectors. (A-B) RNAseq was carried out as described in Figure 4. Principal component analyses (PCA) of RNAseq data were visualized. (C) Volcano plots show up-regulated (Up), down-regulated (Down), or not-significant (N.S.) genes in RNAseq analyses of bulk, MACS-sorted, and cultured cells. (D) NR2F6 KD B16F10 and YUMM1.7 cells were treated with IFNγ (1 ng/ml) for 15 min. Lysates were then immunoblotted for STAT1 phosphorylation, total STAT1 protein, and NR2F6 protein. (E) NR2F6 KD B16F10 cells were treated with IFNγ (1 ng/ml) for indicated times, and STAT1 phosphorylation, total STAT1 protein, and NR2F6 protein were assessed as in (D). (F) Expression of 4 NRs, including NR2F6 was assessed in human melanoma lines treated with IFNγ (left) or TNFα (right) (44). (G) Expression of NR2F6 and NACC1 in CCLE (cancer cell encyclopedia) data from human melanoma lines (*60*), and in human melanoma tissues (TCGA), human melanoma cell culture, and PDX tissues from human melanoma patients. Pearson correlation coefficient (*r*) and corresponding p values (*p*) are shown.

Supplementary figure S5.

NACC1 and FKBP10 are downstream NR2F6 effectors. (A) YUMM1.7 cells were transduced with scrambled (Scr), or two shRNAs (sh194 and sh226) against NR2F6 and selected by blasticidin treatment. The expression of indicated genes was assessed by qPCR. n=3 for each group. (B) CXCL10 expression, as assessed in indicated mouse melanoma cells. n=3 for each group. (C) ReMap peaks predict *Fkbp10* transcriptional regulatory regions. Two candidates, R1 and R2, containing the promoter and intronic *Fkbp10* sequence are predicted. (D) The abundance of NR2F6, RNA polymerase II and H3K27 acetylation on candidate regions was assessed by Chip-qPCR using corresponding antibodies and primers. Relative abundance to input (5% of pre-pulldown material) was calculated. n=3 for each group. (E) The abundance of NR2F6, RNA polymerase II and H3K27 acetylation on R1, was assessed in control and NR2F6 KO B16F10 cells. n=3 for each group. (F) The abundance of NR2F6 binding on R4 region of *Nacc1* was assessed in B16F10 cells overexpressing empty plasmids (pLX304), NR2F6 WT, or NR2F6 MT (DNA-binding mutant, C112S). n=3 for each group. (G) The expression of NR2F6 and NACC1 was assessed in B16F10 cells in (F). (H-I) NR2F6 expression, as assessed in BRAF or NRAS mutant human

melanoma lines by qPCR (F). n=3 for each group. NR2F6 expression levels were compared in melanoma with BRAF or NRAS mutations (G). n=15 (BRAFmt) and n=13 (NRASmt) for each group. (J-K) As in (F-G), NR2F6 expression was assessed in PDX tissues from human melanoma. (L) Expression of NR2F6 and its effectors, NACC1 and FKBP10, as assessed in human melanoma lines as in (H). n=3 for each group. (M-N) Cells of human melanoma lines A375 and Lu1205 were transduced with scrambled (Scr), NR2F6 shRNAs (sh660, sh661, sh662, sh663), and then NR2F6, NACC1, and FKBP10 expression was assessed by qPCR (M), and immunoblotting (N). n=3 for each group. (O-P) Growth of cells transduced with indicated shRNAs for NACC1 (O) and FKBP10 (P), as assessed using CellTiter-Glo. Fold differences in luminescence on days 1 and 3 were calculated relative to luminescence on day 0, defined arbitrarily as 1. n=6 for each group. (Q) The abundance of immune cell types in $CD45^+$ cells, as assessed by FACS using indicated surface markers [B cells ($B220^+$), macrophages ($CD11B^+F4/80^+$), MDSCs ($CD11b^+Gr1^+$), dendritic cells ($CD11c^+MHCII^+$), and NK cells ($NK1.1^+$)]. (R) The abundance of T cells producing indicated cytokines in $CD45^+$ cells, as assessed by FACS. (S) The abundance of exhausted T cells in $CD45^+$ cells, as assessed by FACS. n=5 mice for each group (Q to S). Data are presented as means ± SD. Statistical significance was assessed by Student's t-test (I, K), one-way ANOVA with Dunnett's test (A, B, F), two-way ANOVA with Dunnett's test (O, P), multiple t-tests with Holm-Sidak's test (Q, R, S).

Supplementary figure S6.

NACC1 and FKBP10 are downstream NR2F6 effectors in human cancer. (A) NR2F6, NACC1, and FKBP10 expression in human primary invasive breast tumors from 29 patients, as assessed by scRNAseq (n=51,743 cells), using pre-treated 58 samples (*45*). Shown are average expression (Z-score) and percent expression in malignant cells from responders (E = expanders), and non-responders (NE=non-expanders). (B-C) A Pearson correlation between NR2F6 and NACC1 or FKBP10 was assessed using bulk-RNAseq data from primary pancreatic tumors derived from 224 patients. (D) Differences in survival based on the expression of NACC1, FKBP10, or NR2F6, as assessed using Kaplan-Meier plots of the same patient cohorts (n=222). Day elapsed indicates the days from diagnosis to follow-up census. (E) NR2F6 and its downstream effectors NACC1 and FKBP10 RNA expression log2 (CPM) in tumor tissue and morphologically normal adjacent tissue

(NAT) from lung cancer patients. (F) Kaplan-Meier analysis of lung cancer patients stratified by NR2F6, NACC1, and FKBP10 mRNA levels in the primary tumor, which were greater (green) or less (red) than the median. (G) Scatter plots showing the Pearson correlation between NR2F6 and its downstream effectors NACC1 and FKBP10 expression in early-stage lung cancer tumor tissue. CPM; counts per million reads mapped. n=64 for each group (E to G). Statistical significance was assessed by Student's t-test (E) and log-rank test (D, F).

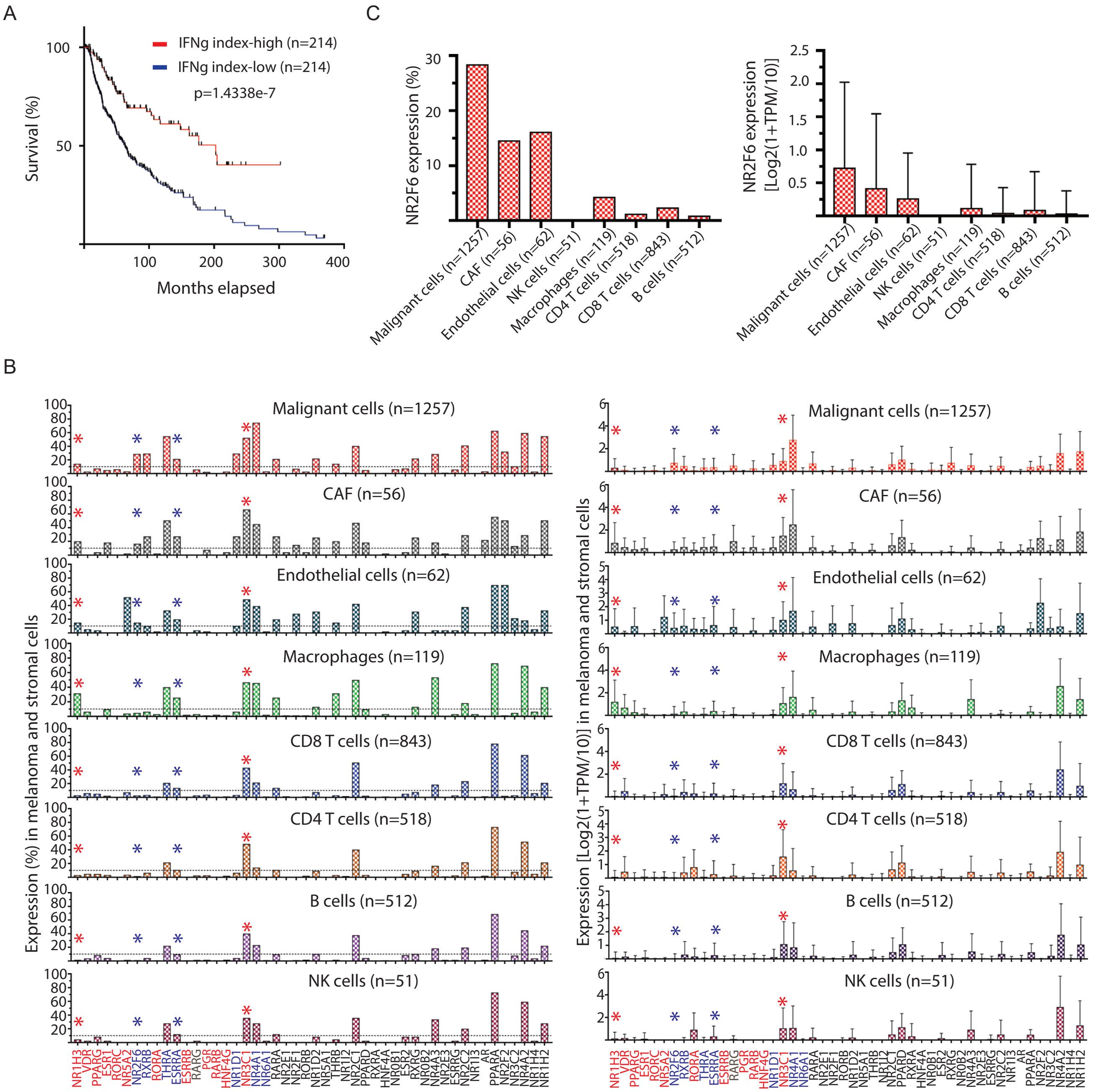

Supplementary figure S1. Kim et al.

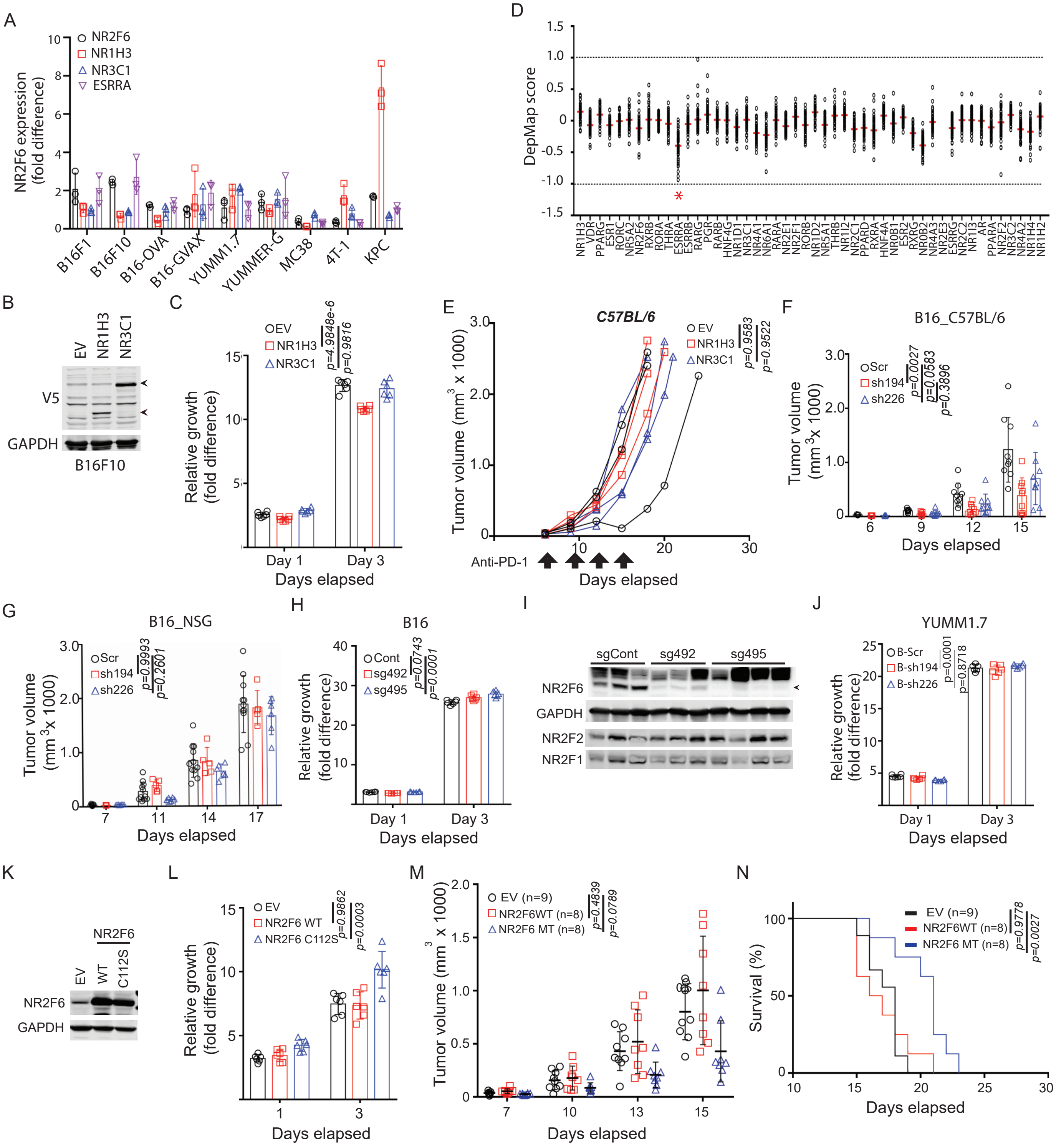

Supplementary figure S2. Kim et al.

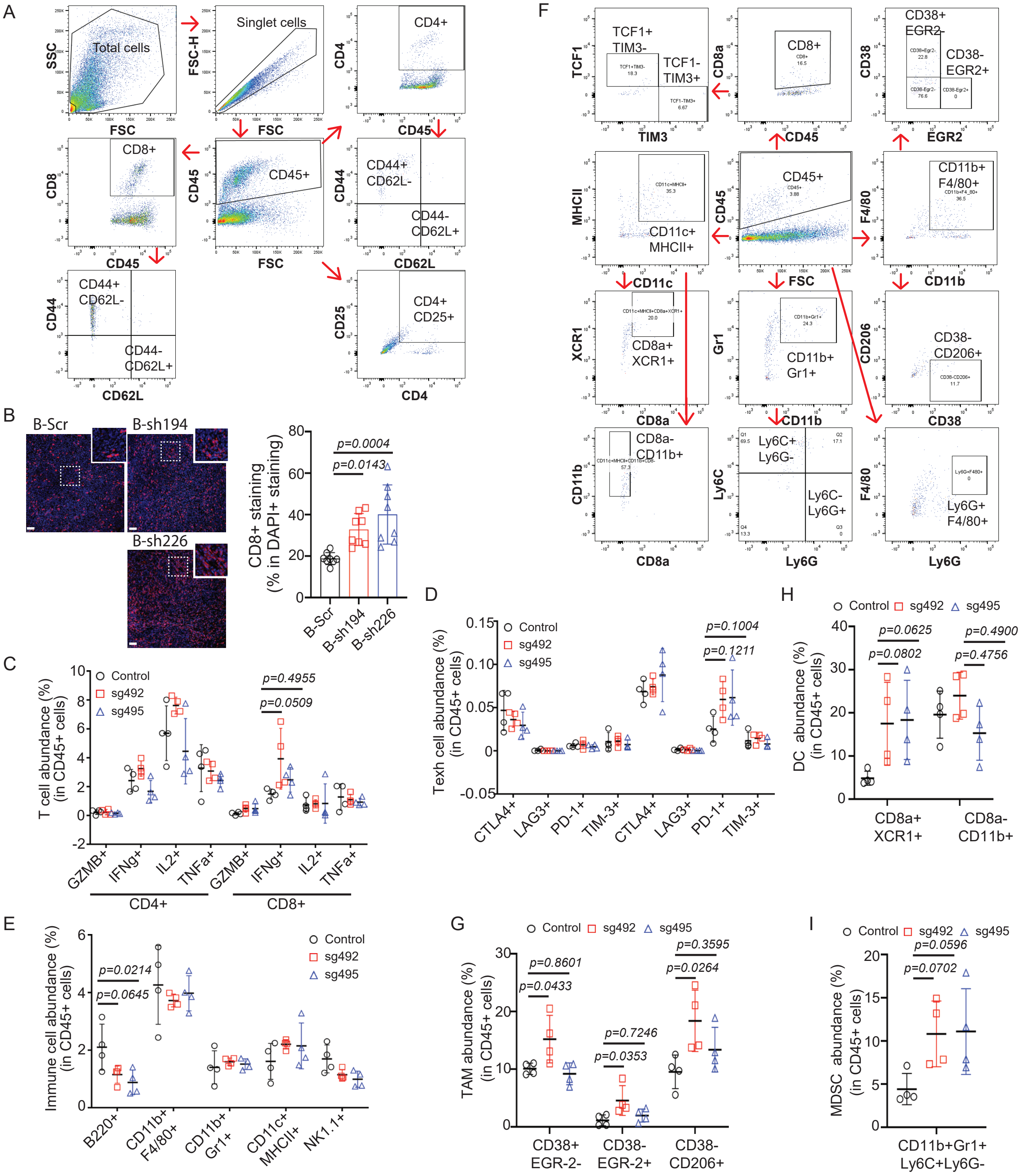

Supplementary figure S3. Kim et al.

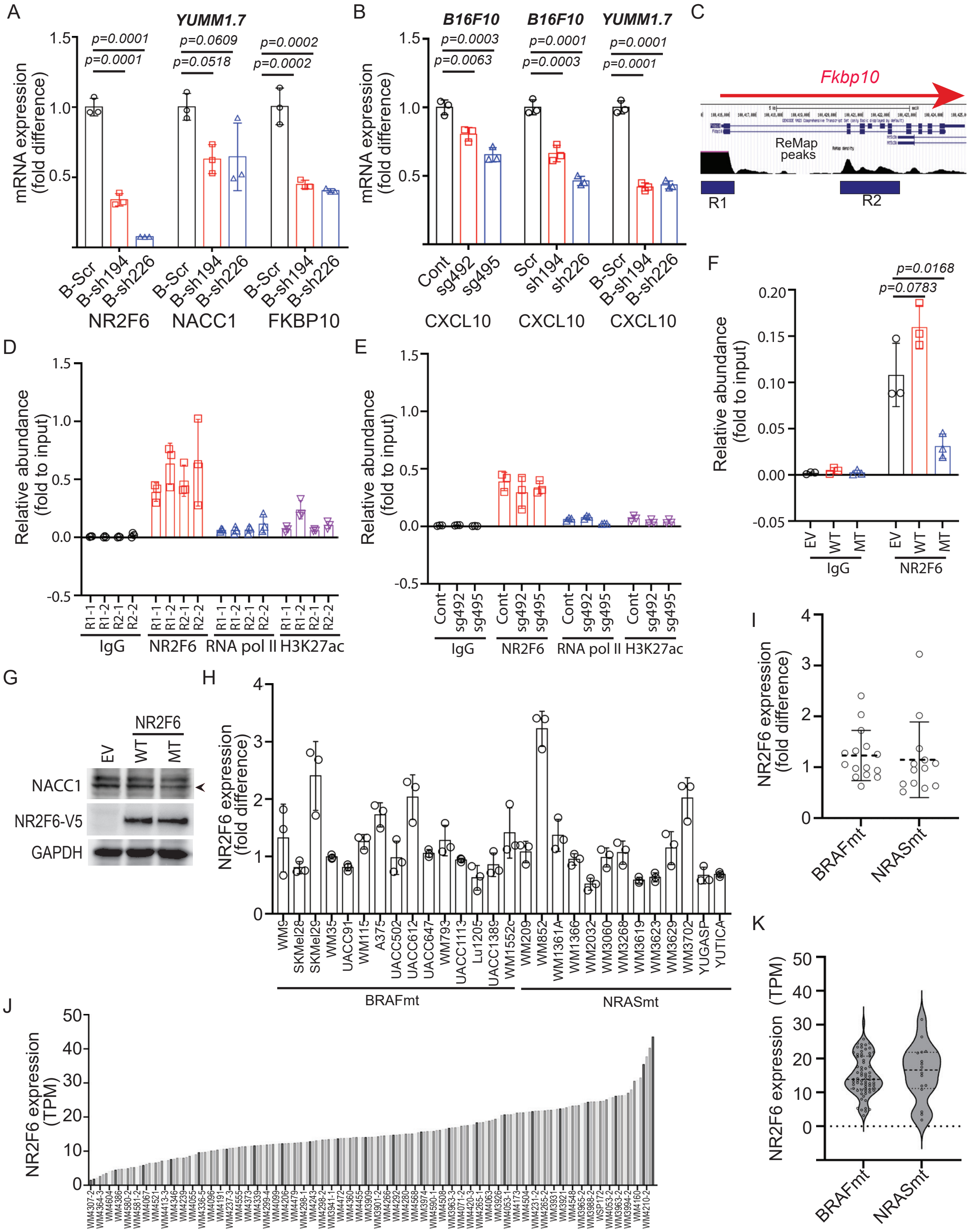

Supplementary figure 5. Kim et al.

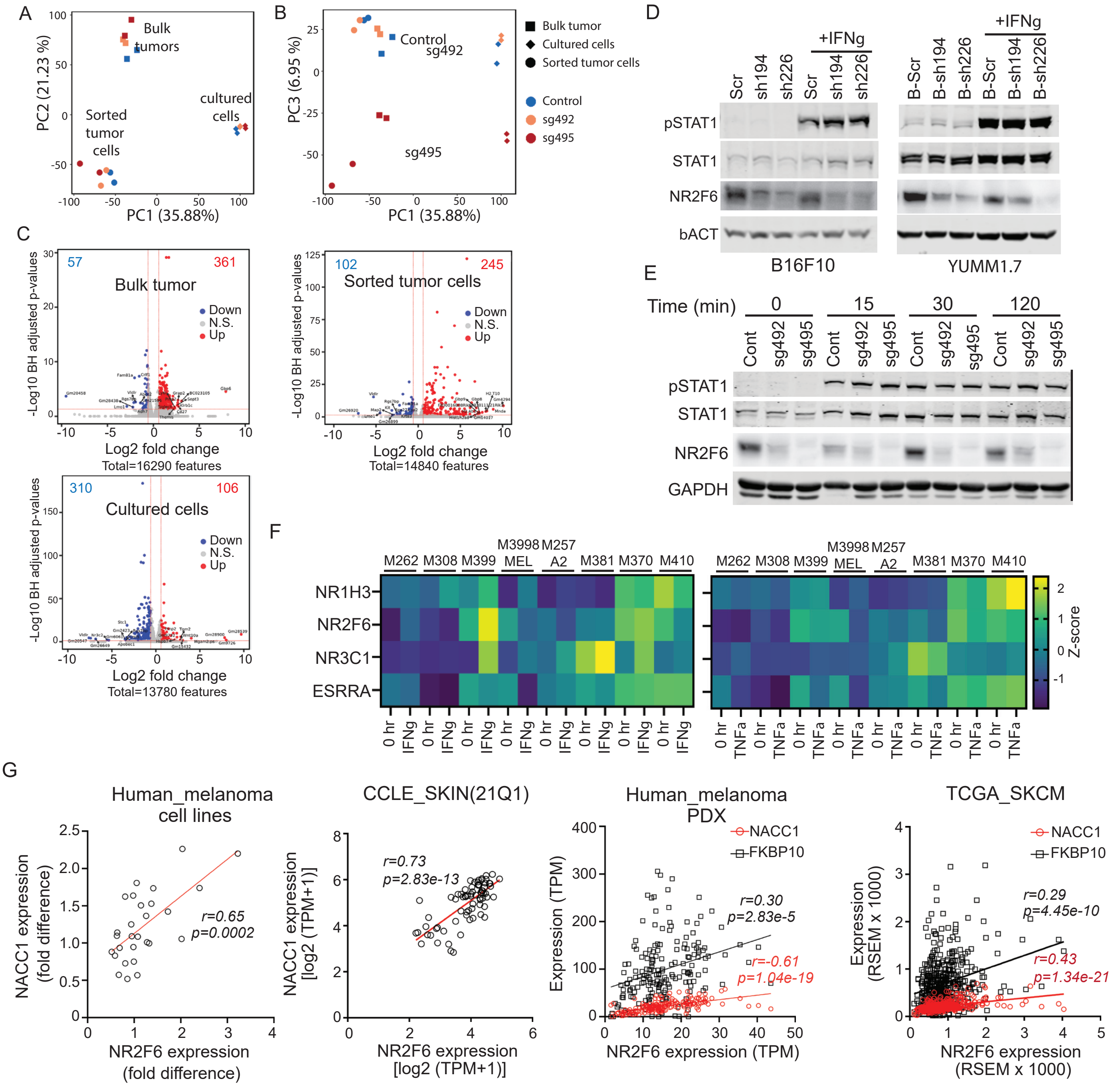

Supplementary figure S4. Kim et al.

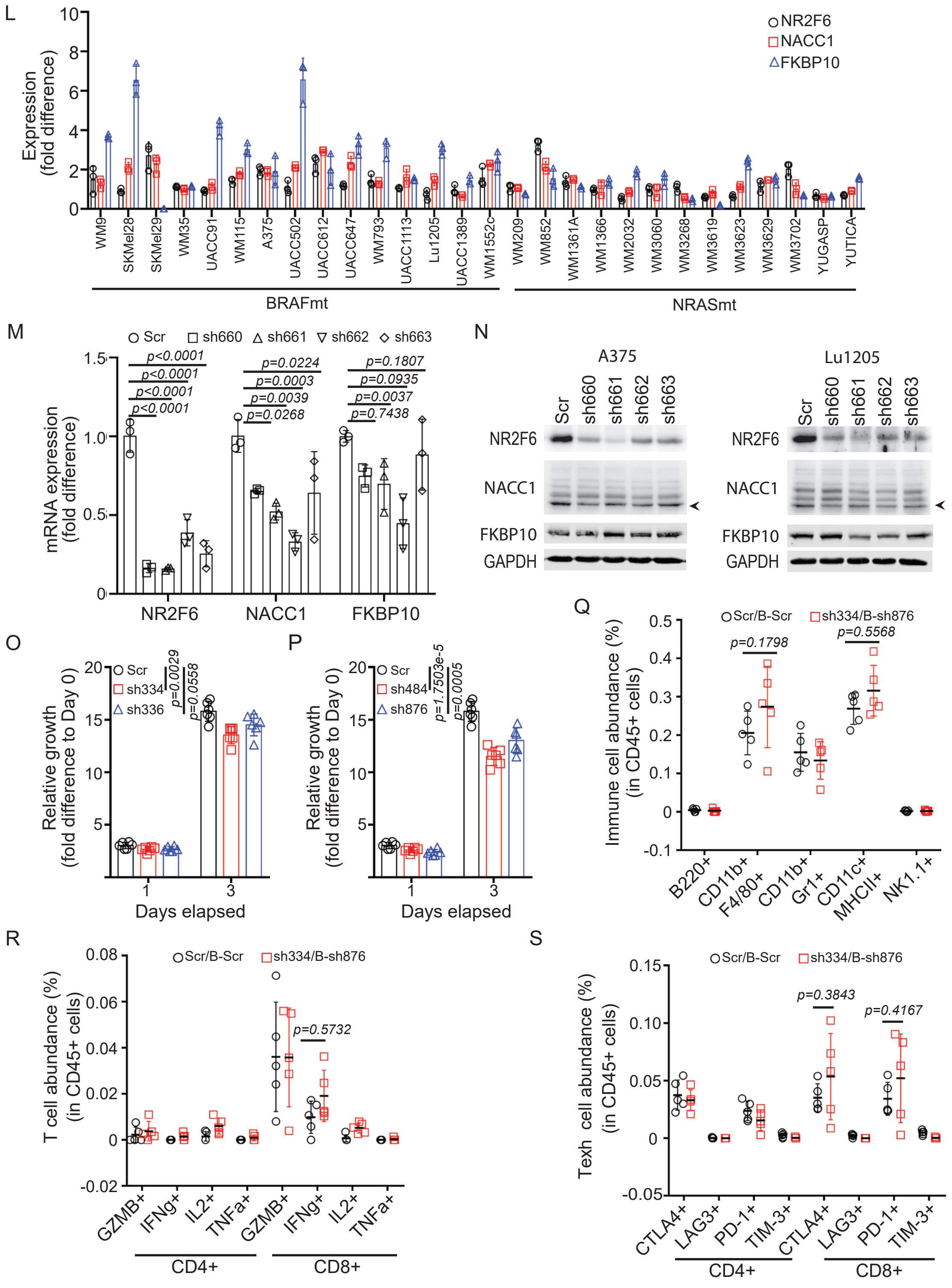

Supplementary figure S5. Kim et al.

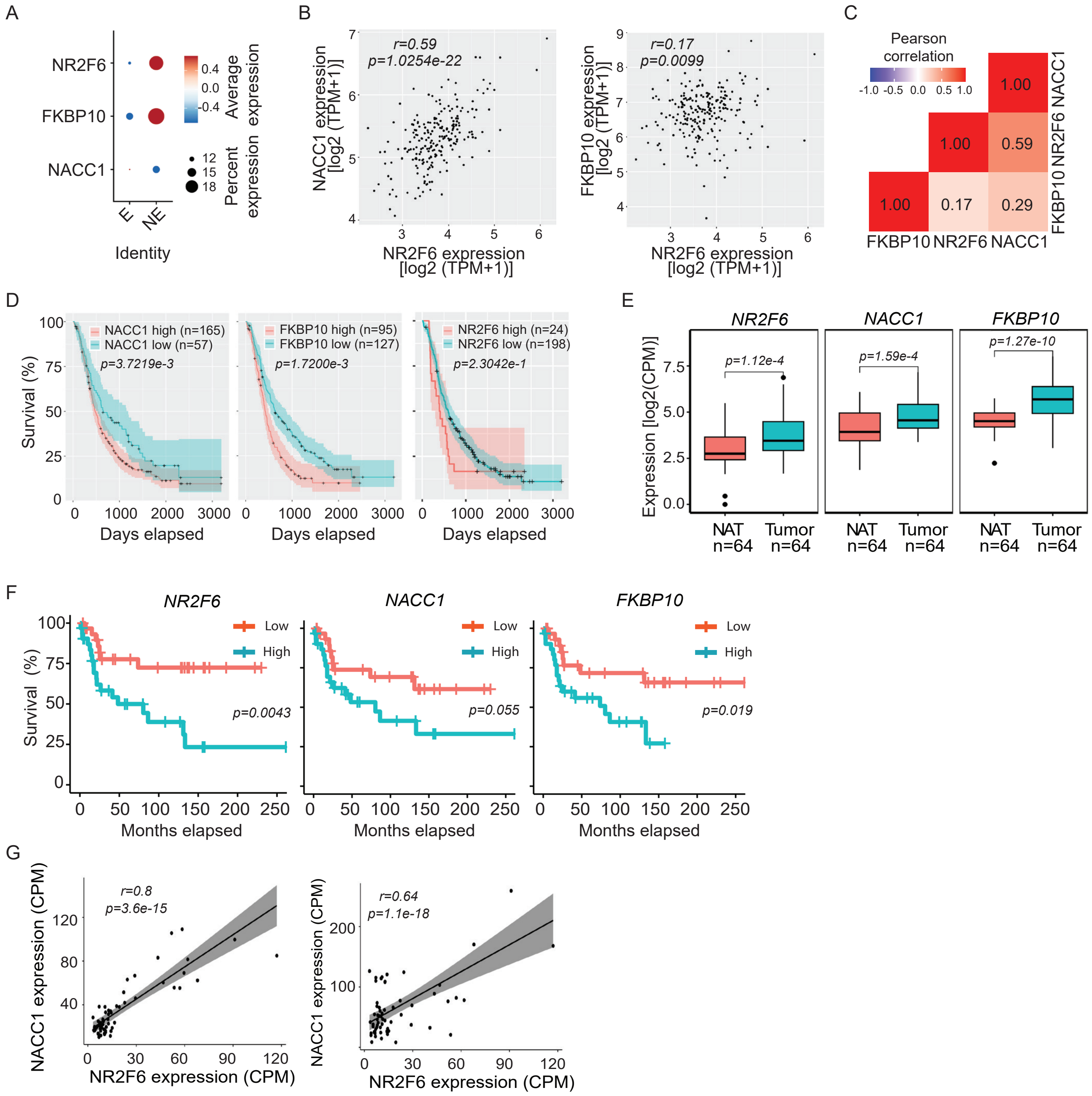

Supplementary figure S6. Kim et al.
